# Supplementary material for: Porous isoreticular non-metal organic frameworks
Source: Nature. 2024 May 22;630(8015):102–8. doi: 10.1038/s41586-024-07353-9 (PMC11153147; doi:10.1038/s41586-024-07353-9)
Supplement: Supplementary file 1 — This file contains Supplementary Methods, 36 Supplementary Figures and 8 Supplementary Tables. [file 41586_2024_7353_MOESM1_ESM.pdf]

nature portfolio

https://doi.org/10.1038/s41586-024-07353-9

Supplementary information

Porous isoreticular non-metal organic frameworks

In the format provided by the
authors and unedited

Porous isoreticular non-metal organic frameworks

Megan O'Shaughnessy,[1] Joseph Glover,[2] Roohollah Hafizi,[2] Mounib Barhi,[3] Rob Clowes,[1] Samantha Y. Chong,[1,4] Stephen P. Argent,[5] Graeme M. Day[2*] and Andrew I. Cooper[1,4*]

[1]Materials Innovation Factory and Department of Chemistry, University of Liverpool, Liverpool, L7 3NY, UK. [2]Computational System Chemistry, School of Chemistry, University of Southampton, Southampton, SO17 IBJ, UK. [3]Albert Crewe Centre for Electron Microscopy, University of Liverpool, Liverpool, L69 3GL, UK. [4]Leverhulme Research Centre for Functional Materials Design, University of Liverpool, Liverpool, L7 3NY, UK. [5]School of Chemistry, University of Nottingham, University Park, Nottingham, NG72 RD, UK.

Supplementary Information

Pg. 2–3 **Section 1. Materials and methods**

Pg. 3–19 **Section 2. Synthesis and characterisation of bulk salts**

Pg. 20–21 **Section 3. Single crystal refinement details**

Pg. 22–27 **Section 4. Iodine capture experiments**

Pg. 28–36 **Section 5. Crystal structure prediction**

Pg. 36–37 **Section 6. Supplementary references**

Section 1. Materials and methods

Materials

All reagents were obtained from Sigma-Aldrich, Manchester Organics, Fluorochem and Alfa Aesar and used as received. All gases for sorption analysis were supplied by BOC at a purity of ≥ 99.9%

NMR

^{1}H NMR spectra were recorded at 400 MHz on a Bruker Advance 400 NMR spectrometer. Chemical shifts are reported in ppm with reference to internal residual protonated species of the deuterated solvents used for ^{1}H analysis.

Thermogravimetric analysis (TGA)

Thermogravimetric analysis was carried out using a Q5000IR (TA Instruments) with an automated vertical overhead thermobalance. Samples (3–5 mg) were heated in platinum pans at a rate of 10 °C min^{-1} unless stated otherwise.

Gas sorption analysis

Nitrogen isotherms were collected at 77 K using an ASAP2420 volumetric adsorption analyser (micrometrics instruments Corporation). Carbon dioxide isotherms were collected up to a pressure of 1200 mbar on a micrometrics ASAP2020 volumetric adsorption analyser at 273 K. Carbon dioxide isotherm at 195 K were collected using Micromeritics 3flex volumetric adsorption analyser. See **Methods**, main text, for further details.

Scanning electron microscopy

The salt samples were dispersed in chloroform at a concentration of 1mg mL^{-1} before being drop cast onto copper grids. TEM images were obtained using a JEOL 2100+ microscope operating at 200KV and equipped with a Gatan Rio Camera.SEM images were collected using a Hitachi S-4800 cold field emission scanning electron microscope. Samples were coated with a 2 nm layer of gold using an Emitech K550X automated sputter coater. Imaging was

conducted at a working voltage of 3 kV and a working distance of 8 mm using a combination of upper and lower secondary electron detectors.

Calculation of pore volumes from crystal structures

Solvent accessible pore volumes, as a fraction of the unit cell volume, were calculated using the “Void” tool in Olex2 (ref. 72, main text) using a probe radius of 1.2 Å and a grid spacing of 0.3 Å.

Section 2. Synthesis and characterisation of bulk salts

Synthesis of bulk crystalline TAPM.Cl

TAPM (0.1 g) was dissolved in acetone (20 mL) and methanolic HCl (1.25 M, 0.7 mL) was added dropwise over 1 min to the solution at room temperature with stirring. The reaction mixture was stirred for 1 h before the solid product was filtered off and dried under vacuum to give crystalline **TAPT.Cl_P1** as an off-white powder (95%).

Crystallisation procedure for TAPM.Cl_P1

To grow suitable crystals for x-ray diffraction, **TAPM** (5 mg) was dissolved in aqueous HCl (5 mL, 1.7 M in water). The resulting clear solution was left for 4 weeks at room temperature, after which thin needles formed on the side of the vial. SCXRD data of the crystals revealed that **TAPM.Cl_P1** crystallises in the tetragonal space group $P4_2/nmc$ (Figure 3.1a). This structure is densely packed with no pore channels (Figure 1).

Crystal data for **TAPM.Cl_P1**: Formula $C_{25}H_{28}N_{44}(Cl)$; M = 64.28, Tetragonal $P4_2/nmc$, colourless needle shaped crystals; crystal size = 0.065 x 0.024 x 0.02 mm^3; a = 13.2945(5) Å, b = 13.2945(5) Å, c = 7.5715(4) α,β,γ = 90°, V = 1338.22(12) $Å^3$; ρ = 1.276 g cm^{-3}; μ(rotating anode Mo-Kα λ = 0.71073 Å) = 0.461 mm^{-3}; F (000) = 524; T = 100 K; 5172 reflections measured (2.166 < 2ϴ < 30.161 °), 949 unique (R_{int} = 0.0357), 781 ($I > 2\sigma(I)$); R_1 = 0.0357 for observed and R_1 = 0.0447 for all reflections; wR_2 = 0.1036 for all reflections; max/min difference electron density = 0.459 and -0.223 e.A^{-3}; data/restraints/parameters = 949/0/46; GOF = 1.035. CCDC code; 2308598, see CIF for all refinement details.

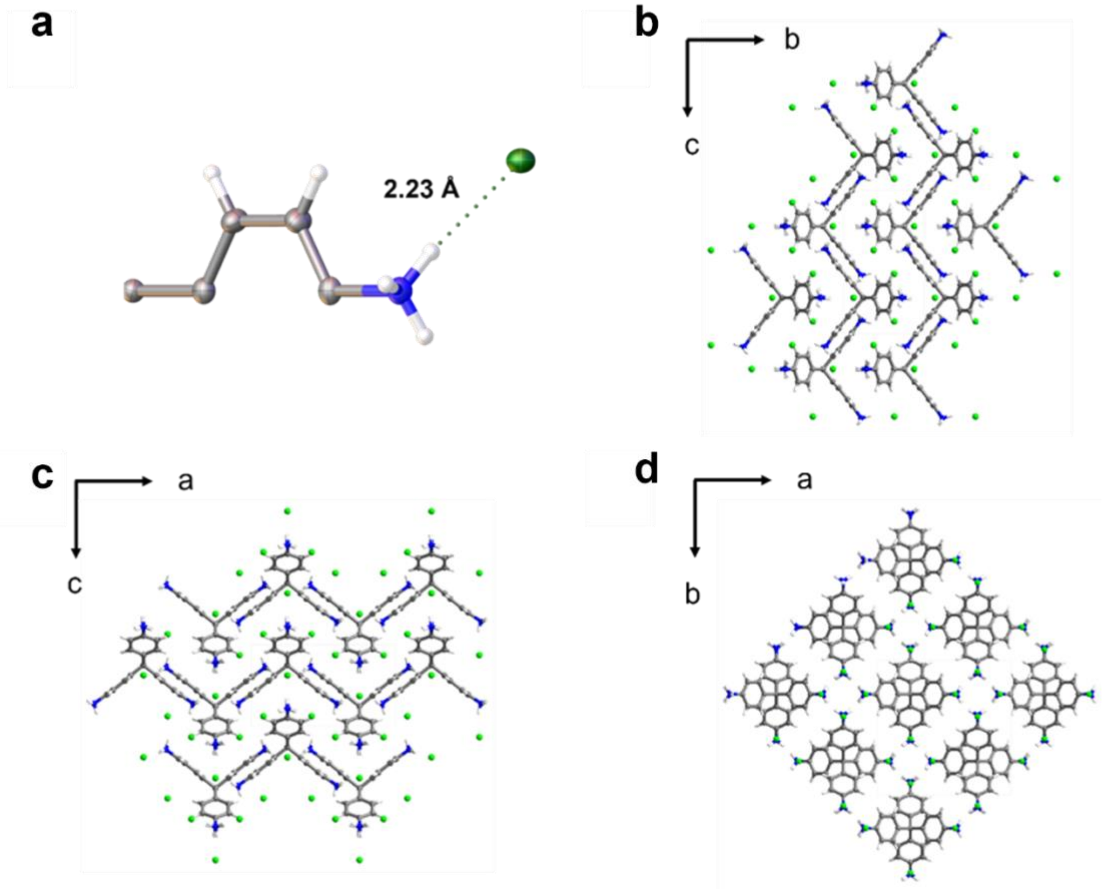

Figure 1. a, Displacement ellipsoid plot from the single crystal structure of **TAPM.Cl_P1**. Ellipsoids are displayed at 50% probability. Atom labels are omitted for clarity. **b–d**, Crystal packing of **TAPM.Cl_P1** viewed along the *a*, *b,* and *c* crystallography axis respectively. grey: carbon; red: oxygen; blue: nitrogen green: chlorine; white: hydrogen.

Crystallisation procedure for TAPM.Cl_P2

Powdered crystalline **TAPM.Cl** (4 mg) was added to chlorobenzene (0.1 mL) followed by MeOH (0.5 mL) giving a clear solution. The solution was then heated at 60 ºC for 2 h resulting in thin, long red needles of **TAPM.Cl_P2**. SCXRD revealed that **TAPM.Cl_P2** crystallises in the monoclinic space group *C12/c1* (Figure 2). Again, like **TAPM.Cl_P1** this structure is a non-porous, densely packed salt (Figure 2b-d)

Crystal data for **TAPM.Cl_P2**: Formula: 3.2(Cl), 0.8(C_{25} H_{28} N_4), 1.6(C H_4 O), 0.2(O): M = 529.51, Monoclinic *C12/c1*, red colourless needle shaped crystals; crystal size = 0.069 x 0.027 x 0.02 mm^3; a = 12.4607(4) Å, b = 20.2943(7) Å, c = 11.9466(4) Å, β = 101.801(4) °, V = 2957.220 Å^3; ρ = 1.487 g cm^{-3}; μ(rotating anode Mo-Kα λ = 0.71073 Å) = 0.524 mm^{-3}; *F* (000) = 524; T = 100 K; 10396 reflections measured (2.390 < 2ϴ < 60.746 °), 3589 unique (R_{int} = 0.0357), 781 ($I > 2\sigma(I)$); R_1 = 0.0240 for observed and R_1 = 0.0755 for all reflections; wR_2 = 0.2007 for all reflections; max/min difference electron density = 1.032 and -0.829 e.A^{-3};

data/restraints/parameters = 3589 /0/176; GOF = 1.067. CCDC code; 2308599, see CIF for all refinement details.

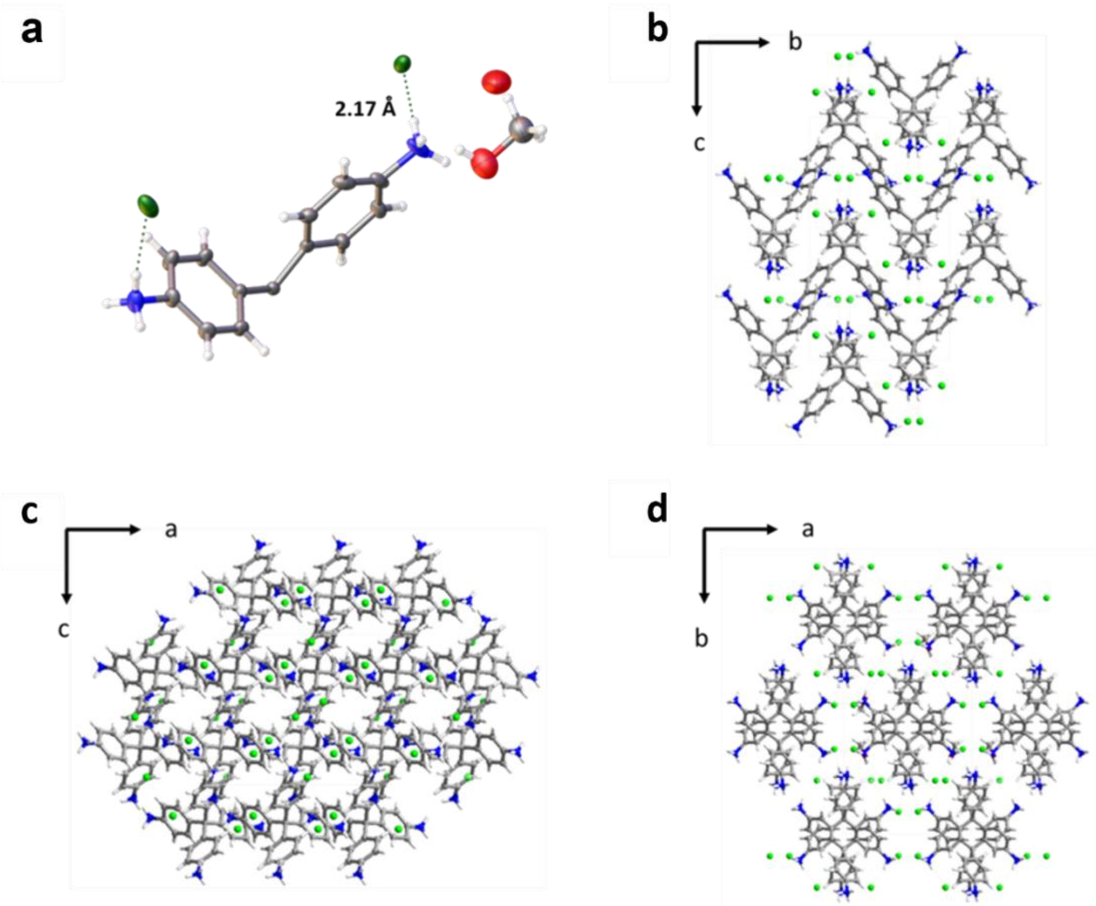

Figure 2. **a**, Displacement ellipsoid plot from the single crystal structure of **TAPM.Cl_P2**. Ellipsoids are displayed at 50% probability. Atom labels are omitted for clarity. **b–d**, Crystal packing of viewed along the *a*, *b,* and *c* crystallography axis respectively. Solvent molecules have been omitted for clarity. grey: carbon; red: oxygen; blue: nitrogen green: chlorine; white: hydrogen.

Synthesis of bulk crystalline TAPM.Br

TAPM (0.1 g) was dissolved in acetone (20 mL) and HBr (0.15 mL, 48 wt. % in water) was added dropwise over 1 min to the solution at room temperature with stirring. The reaction mixture was stirred for 1 h before the solid product was filtered and dried under vacuum to give crystalline **TAPT.Br_P1** as an off-white powder (97%).

Crystallisation procedure for TAPM.Br_P1

Single crystals of **TAPM.Br** suitable for x-ray diffraction were grown by dissolving **TAPM** (5 mg) in aqueous hydrobromic acid (3 mL, 48 wt. % in water) with heating at 80 ºC. After

heating for 6 h, the resulting clear solution was cooled to room temperature and left open to the air. After 2 days, a large block of single crystals of **TAPM.Br_P1** was formed. SCXRD revealed that **TAPM.Br_P1** crystallises into the triclinic space group *P* (Figure 3a). The structure was a non-porous, densely packed salt (Figure 3b-d).

Crystal data for **TAPM.Br_P1**: Crystal data for TAPM.Br_P1: Formula: C_{25} $H_{27.25}$ N_4, 4(Br); M =238.78, Triclinic *P*, brownish colourless block shaped crystals; crystal size = 0.2 x 0.2 x 0.23 mm^3; a = 11.2877(5) Å, b = 11.7704(5) Å, c = 11.9638(5) Å, A = 90.265(3)° β = 107.249(4)°, γ = 114.896(4) °, V = 1361.50(11) Å^3; ρ = 1.718 g cm^{-3}; μ(rotating anode Mo-Kα λ = 0.71073 Å) = 4.439 mm^{-3}; F (000) = 692; T = 113 K; 16628 reflections measured (2.106 < 2Θ < 30.356°), 6616 unique (R_{int} = 0.0319), 5147 ($I > 2\sigma(I)$); R_1 = 0.0322 for observed and R_1 = 0.0503 or all reflections; wR_2 = 0.0503 for all reflections; max/min difference electron density = 0.855 and -0.854 e.A^{-3}; data/restraints/parameters = 6616/0/302; GOF = 1.034. CCDC code; 2308596, see CIF for all refinement details.

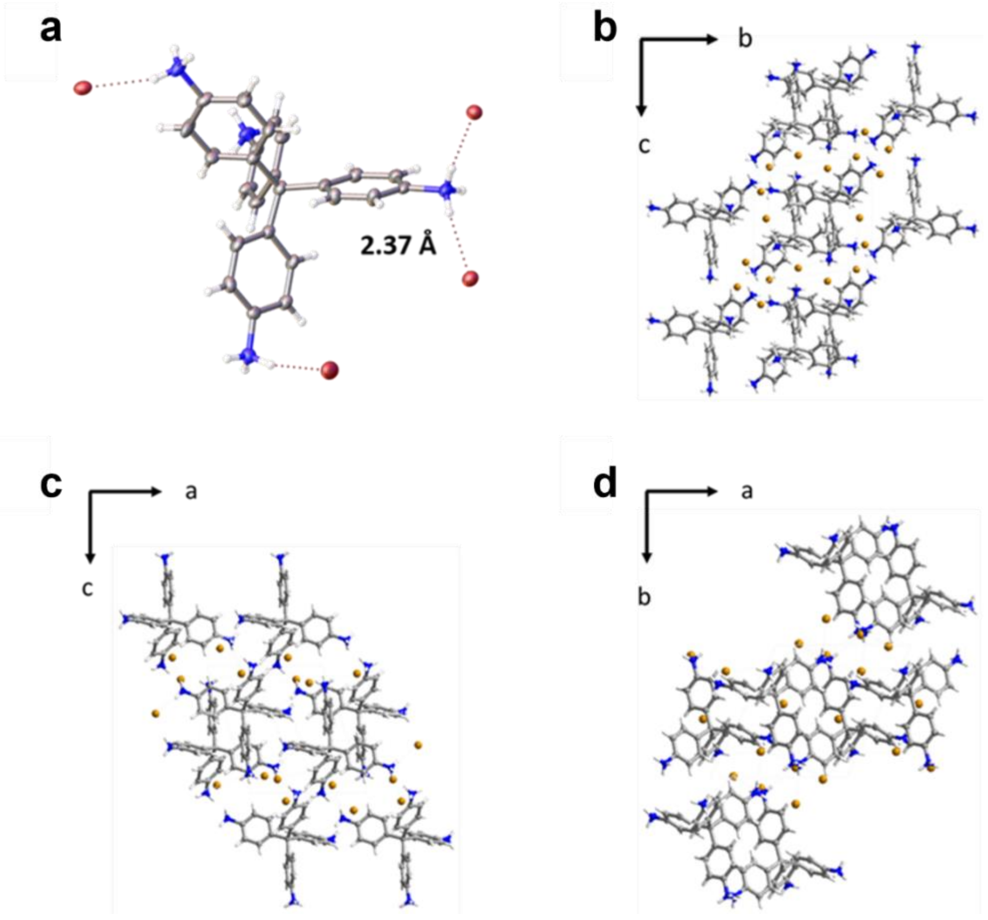

Figure 3. **a**, Displacement ellipsoid plot from the single crystal structure of **TAPM.Br_P1**. Ellipsoids are displayed at 50% probability. Atom labels are omitted for clarity. **b–d**, Crystal

packing of **TAPM.Br_P1** viewed along the *a*, *b,* and *c* crystallography axis respectively. Solvent molecules have been omitted for clarity. grey: carbon; red: oxygen; blue: nitrogen; brown: bromine; white: hydrogen.

Crystallisation procedure for TAPM.Br_P2

Single crystals of **TAPM.Br_P2** suitable for x-ray diffraction were grown by dissolving TAPM.Br in MeOH (0.2 mL) and adding the solution to benzonitrile (0.1 mL). The clear solution was then heated at 60 °C to evaporate the MeOH evaporating over 30 minutes, which left long, thin needles of **TAPM.Br_P2** in the remaining benzonitrile. CCDC code; 2308597, see CIF for crystal and refinement details. The crystal structure for **TAPM.Br_P2** was isostructural with **TAPM.Cl_P2** (see Extended Data Fig. 1 for a comparison of the two crystal packings); the similarities in their unit cells are summarised in Table 1.

Table 1. Cell parameters for the isostructural chloride (**TAPM.Cl_P2**) and bromide (**TAPT.Br_P2**) salts of TAPM.

	**Space group**	**α (Å)**	***b*** **(Å)**	***c*** **(Å)**	**β (°)**	***V*** **(Å^3)**
**TAPM.Cl_P2**	*C*12/*c*1	12.46	20.29	11.94	101.80	2957.22
**TAPM.Br_P2**	*C*12/*c*1	12.84	21.04	12.17	101.28	3225.14

Synthesis of bulk crystalline TAPT.Cl

Bulk powders of **TAPT.Cl** were formed by dissolving TAPT (2 g) in an effective solvent (see Table 2 below for list of 60 solvents investigated) at a concentration of 5 mg mL^{-1}. Methanolic HCl was then added dropwise over 5 min at room temperature with stirring. The mixture was stirred for a further 1 h before the solvent was removed under reduced pressure and the resulting solid was washed with THF, giving crystalline **TAPT.Cl** as an off-white solid (2.57 g, 98%).

Table 2. Solubility tests for **TAPT** in a range of 60 different organic solvents; the solvents in the darker orange cells gave a solubility > 5 mg mL^{-1}; the solvents in the lighter orange cells gave a solubility 1–5 mg mL^{-1}. The uncoloured entries in the table refer to solvents that did not dissolve the TAPT appreciably. These same 60 solvents were evaluated for all the amine linkers. HFP = hexafluoropropanol, DCM = dichloromethane, THF = tetrahydrofuran, DMF = dimethylformamide, DMA = Dimethylacetamide, DEF = diethylformamide, DMSO = dimethylsulfoxide, NMP = *N*-methylpyrrolidone.

Diethyl ether	Pentane	DCM	Acetone	Methyl acetate
HFP	Chloroform	Methanol	THF	Hexane
Diisopropyl ether	1,3-Dioxolane	Ethyl acetate	Trifluoroethanol	Ethanol
Cyclohexane	Acetonitrile	2-Propanol	2,2 -Dimethoxypropane	Tetrahydropyran
1-Propanol	n-heptane	Water	1,4-Dioxane	1,4-Difluorobenzene
Hexafluorobenzene	1-Butanol	Cyclopentanone	Chlorobenzene	Toluene
DMF	Cyclohexanone	m-xylene	p-xylene	o-xylene
DMA	DEF	DMSO	NMP	Mesitylene
γ-Butyrolactone	Butyl Benzene	1,2-Dimethoxybenzene	1,3-Dimethoxybenzene	1,3-Propanediol
Diphenyl ether	Anisole	Methyl tetrahydrofuran (MTF)	tert-butyl methyl ether	N-methyl-morpholine

The effective solvents (dark orange entries) for this material were further narrowed down to focus on those that had either low boiling points and/or were likely to be less coordinating. Our aim was to limit the chance of solvates forming and to minimize difficulties in activating any potentially porous solvates that were formed. Based on these criteria, the focused library of solvents that was chosen was DCM, acetone, methyl acetate (MeOAC), chloroform ($CHCl_3$), THF, ethyl acetate (EtOAc), and methyl tetrahydrofuran (MTF). Apart from DCM and $CHCl_3$, instant precipitation occurred upon addition of HX (X = Cl, Br). After up to two hours of stirring, the solvent either evaporated or the solid was filtered off to give the salt products. PXRD data showed that the same polymorph formed in all seven of the solvent conditions tested (Figure 4). To search other polymorphs, we also evaluated HCl solutions in other solvents such as in

diethyl ether, EtOAc, and dioxane, but these also produced what appeared, qualitatively, to be the same polymorph based on PXRD data

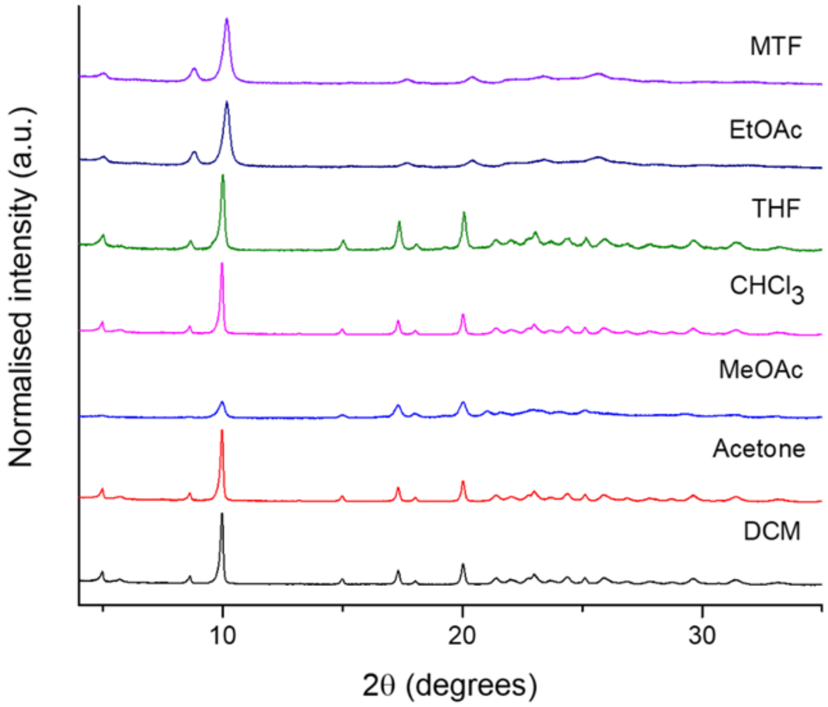

Figure 4. PXRD patterns for bulk crystalline powders of **TAPT.Cl** formed using a range of solvents. These results suggest that the same polymorph is formed in all conditions tested, which could be matched with a low-energy CSP structure (main text, Figure 2a,d,g). The salt appeared to be notably less crystalline when prepared in MeOAc. THF, $CHCl_3$, acetone and DCM appeared to give the most crystalline powders based on qualitative analysis of the PXRD data.

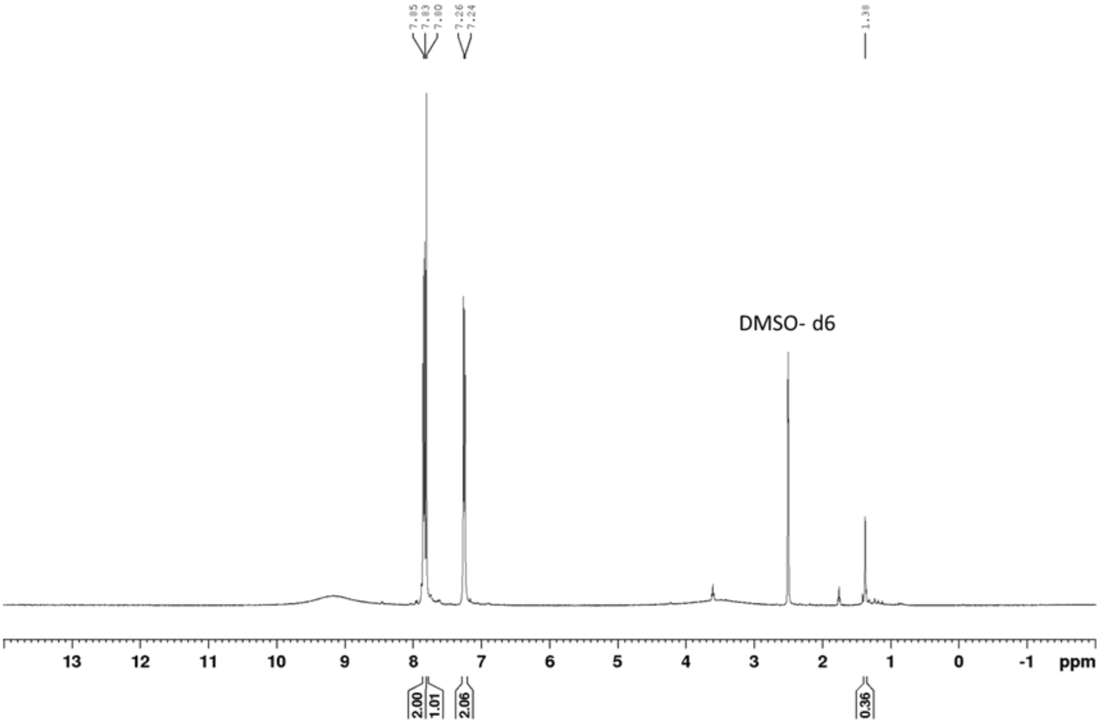

Figure 5. ^{1}H NMR (400 MHz, DMSO) of the product peaks for **TAPT.Cl**. ^{1}H NMR (400 MHz, DMSO) δ 7.8 (s, 1H), 7.83 (d, 2H), 7.24 (d, 2H), 1.37 (s, 2H).

Crystallisation procedure for TAPT.Cl

Single crystals of **TAPT.Cl** suitable for x-ray diffraction were grown by dissolving **TAPT.Cl** (3 mg) in MeOH (0.5 mL) and chlorobenzene (0.05 mL). The solvent was left to evaporate at room temperature over 16 h, which gave block crystals of **TAPT.Cl**. While the crystals were of a sufficient size (Figure 8) they showed weak diffraction to low angles using a laboratory-based diffractometer. To solve the structure, we used synchrotron radiation at the Diamond Light Source. Synchrotron SCXRD revealed that the salt crystallised as a solvate in the trigonal space group (Figure 6a).

The crystal structure contains two different types of channels running parallel to the c-axis; a cylindrical channel bounded by protonated amines and chloride anions (labelled as pore 'A' in Figure 2d) and two inequivalent diamond-shaped channels bound by C-H moieties (labelled as pore 'B' in Figure 2d). All these channels contained residual electron density, but only one of the narrow diamond-shaped channels contained chlorobenzene solvent molecules that could be modelled reasonably. The chlorobenzene molecules are disordered over an inversion centre, resulting in disorder of the chlorine atom in the solvent. SADI and FLAT restraints were used on the disordered chlorobenzene molecules. A solvent mask was used on the other two channels; the electron density in the second diamond-shaped channel was indicative of disordered chlorobenzene residues. The electron density in the much more polar

circular channel (pore A, Figure 2d) was indicative of disordered water molecules. The number of electrons and void volumes were consistent with an average of one chlorobenzene molecule in the diamond-shaped channels (pore B) and 1.5 water molecules in the cylindrical channel (pore A) per asymmetric unit cell. All residues treated with solvent mask are included in the unit cell contents and formula moiety. A SADI restraint was used on the phenyl rings in the TAPT molecule.

Crystal data for **TAPT.Cl**: Formula 3(Cl), C_{24} H_{24} N_3, 1.25[C_6H_5Cl], 1.5[H_2O]; M = 628.52, Trigonal *P3m1*, colourless block shaped crystals; crystal size = 0.02 x 0.02 x 0.01 mm^3; a = 35.3856(2) Å, b = 35.3856(2) Å, c = 8.17500(10) Å α = 90° ,β, = 90°, γ = 120°, V = 8864.85 (15) $Å^3$; ρ = 1.413 g cm^{-3}; μ(synchrotron λ = 0.6889 Å) = 0.422 mm^{-3}; *F* (000) = 3930; T = 100 K; 144458 reflections measured (2.232 < 2ϴ < 54.678°), 7685 unique (R_{int} = 0.0797), 5263 ($I > 2\sigma(I)$); R_1 = 0.0797 for observed and R_1 = 0.0962 for all reflections; wR_2 = 0.2710 for all reflections; max/min difference electron density = 0.706 and -0.440 e.A^{-3}; data/restraints/parameters = 7685/245/329; GOF = 1.116. CCDC code; 2308662, see CIF for all refinement details.

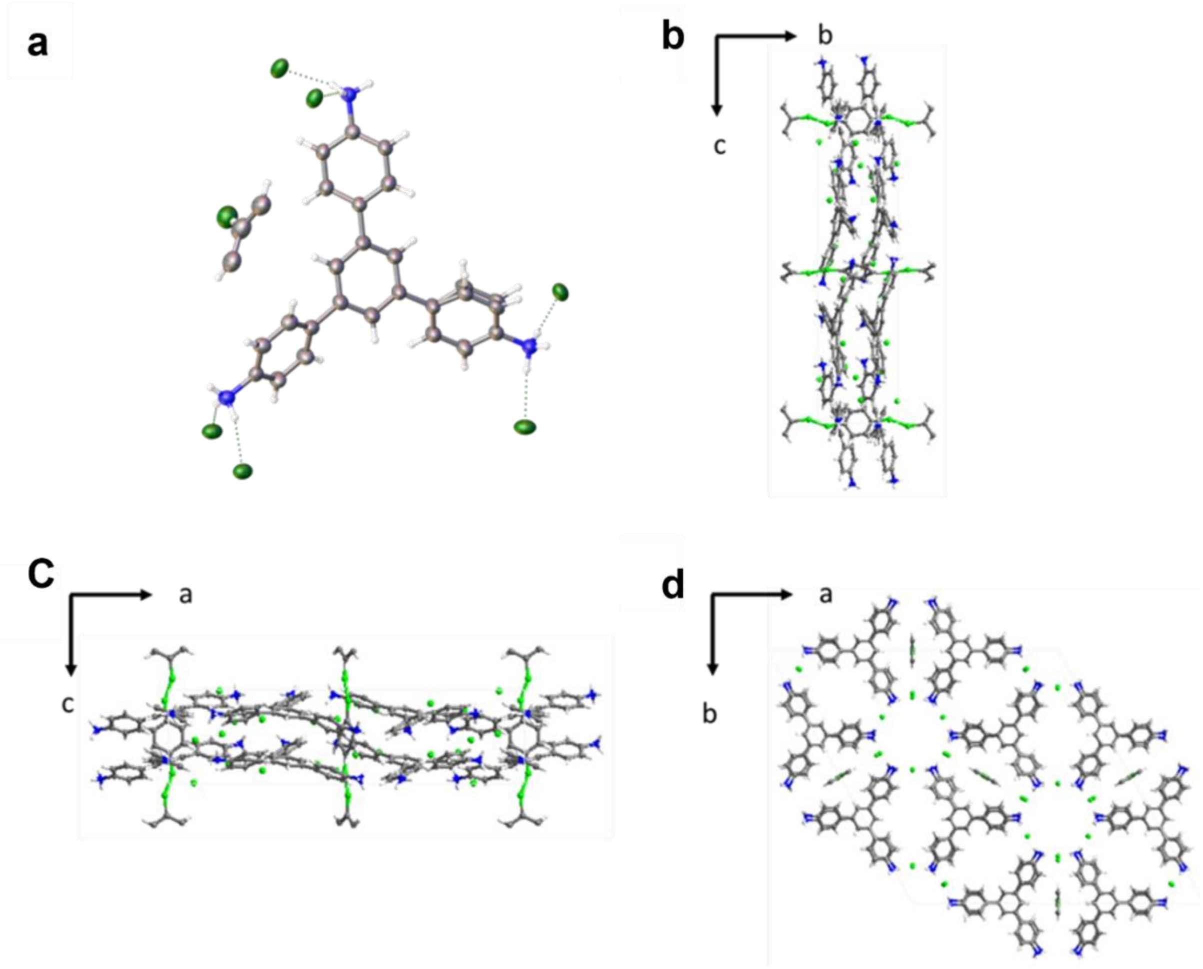

Figure 6. **a**, Displacement ellipsoid plot from the single crystal structure of **TAPT.Cl**. Ellipsoids are displayed at 50% probability. Atom labels are omitted for clarity. **b–d**, Crystal packing of **TAPT.Cl** viewed along the *a*, *b,* and *c* crystallography axis respectively. grey: carbon; red: oxygen; blue: nitrogen green: chlorine; white: hydrogen. Solvent is omitted for clarity.

Synthesis of bulk crystalline TT.Br

The solubility of **TT** was also tested in the solvents listed in Table 2 and it was found to be significantly less soluble than **TAPT**; indeed, the only 'good' solvents (>5 mg mL^{-1}) were acetone, MeOAc, THF, and EtOAc. The solvent that gave powders with the highest crystallinity was THF. The **TT** concentration was 5 mg mL^{-1} and HBr (0.15 mL, 48 wt.% in water on a 0.1 g scale) was added dropwise with stirring and the mixture was left for 1 h before collecting the product (97%).

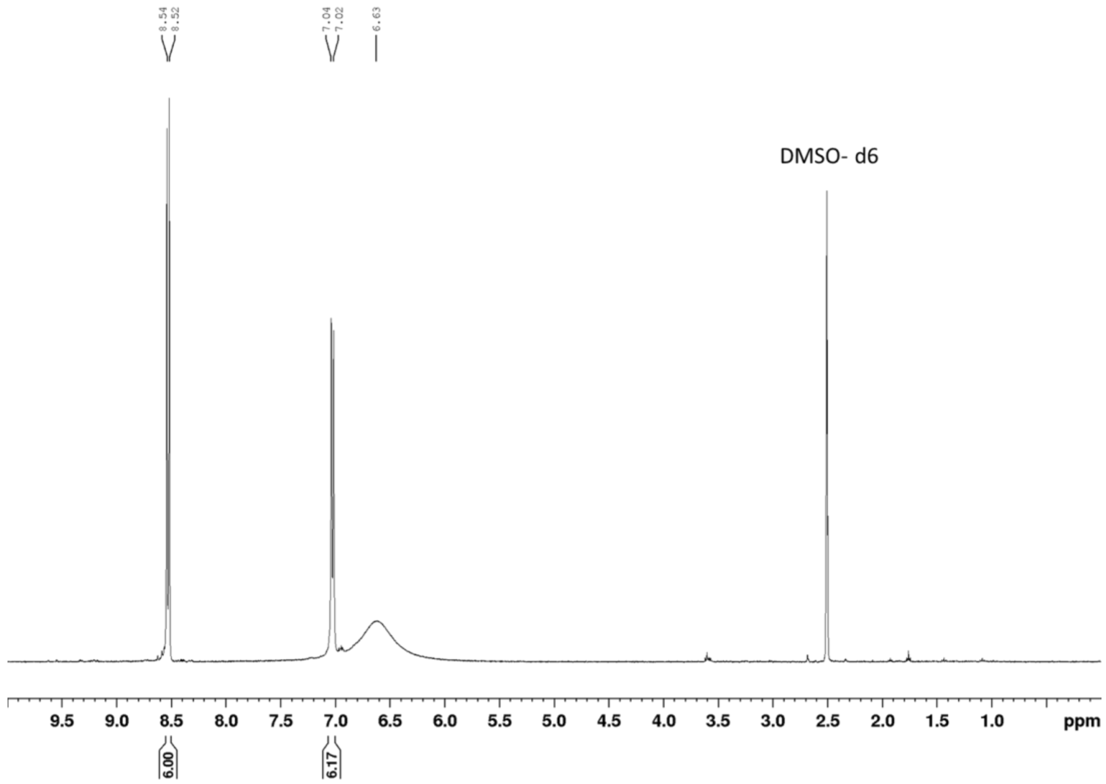

Figure 7. ^{1}H NMR (400 MHz, DMSO) of the product peaks for **TT.Br**. ^{1}H NMR (400 MHz, DMSO) δ 8.53 (d, 6H), 7.03 (d, 6H), 6.63 (s, broad 6H).

Crystallisation conditions for TT.Br

The best crystals obtained for **TT.Br** were obtained using the following conditions: **TT.Br** (3 mg) in benzonitrile (0.1 mL) and MeOH (0.5 mL) were heated at 60 °C for 2 h to give long, thin needles (Figure 8). Synchrotron radiation at the Diamond Light Source was used in an attempt to determine the structure. While the **TT.Br** crystals showed good diffraction initially, they rapidly degraded under synchrotron irradiation before enough data could be collected. Various attempts were made to collect data for these crystals by varying the radiation and collection times, but due to both the small size of the crystals and their sensitivity to radiation damage, solving the structure was impossible. However, unit cells collected during these structure solution attempts showed the material had a similar unit cell to that determined by

SCXRD for **TAPT.Cl**, providing further evidence that these two samples might be isostructural, as predicted (main text, Figure 2). The differences in the crystal sizes of both **TAPT.Cl** and **TT.Br** can be seen in Figure 8.

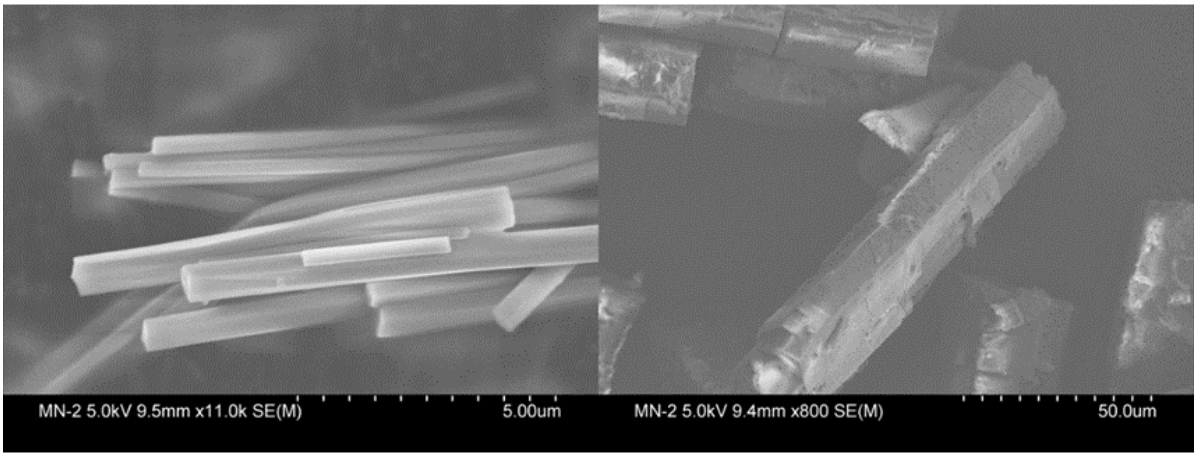

Figure 8. Scanning electron microscope images for the best crystals from the crystallisations of **TT.Br** (left) from benzonitrile/MeOH and **TAPT.Cl** (right) in chlorobenzene/MeOH. Scale bars are included in the bottom right corner of the images; note the much smaller size of the **TT.Br** crystals which, combined with sensitivity to synchrotron beam damage, precluded structure solution by SCXRD.

Crystallisation of TTBT.Cl

The solubility of **TTBT** was tested in the same solvents as **TAPT** and **TT** (Table 2). It was shown to have good solubility (>5 mg mL^{-1}) in acetone, THF, HFIP, and MTF. The same **TTBT.Cl** polymorph was formed in all these solvents, based on PXRD data (Figure 9). Matching these PXRD data against diffraction patterns derived from CSP structures for **TTBT.Cl** indicated that the structure was an extended form of the packing observed for **TAPT.Cl** and **TT.Br**. The match between the experimental PXRD pattern and calculated pattern for the most similar CSP-derived structure is shown in Figure 9. The disagreement in peak intensities may be due to preferred orientation in the experimental samples. Other discrepancies at higher angles could be due, for example, to the CSP methodology not totally capturing the torsion angles in the more torsionally complex biphenyl struts in **TTBT**. This is the most complex system that we attempted by CSP here; it has four independent units (**TTBT** plus three chloride ions) and a significantly larger number of potential conformers for the **TTBT** molecule itself. As such, the structural search space is much larger in terms of the number of degrees of freedom that are available.

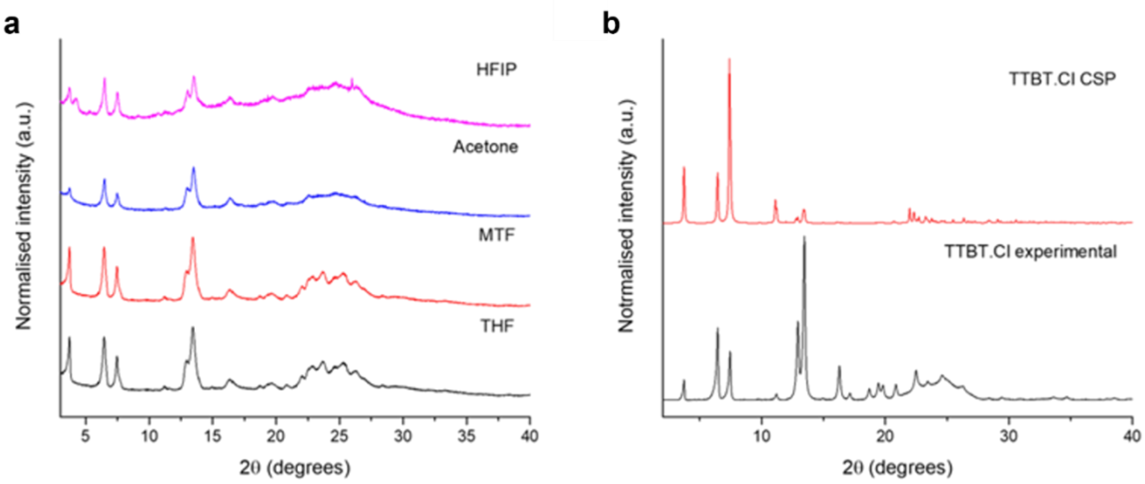

Figure 9. **a**, PXRD patterns for **TTBT.Cl** as obtained under different solvent conditions indicating that the same polymorph was formed in all cases. **b**, PXRD comparison for experimental data and the most closely matched CSP derived data (see Figure 2c).

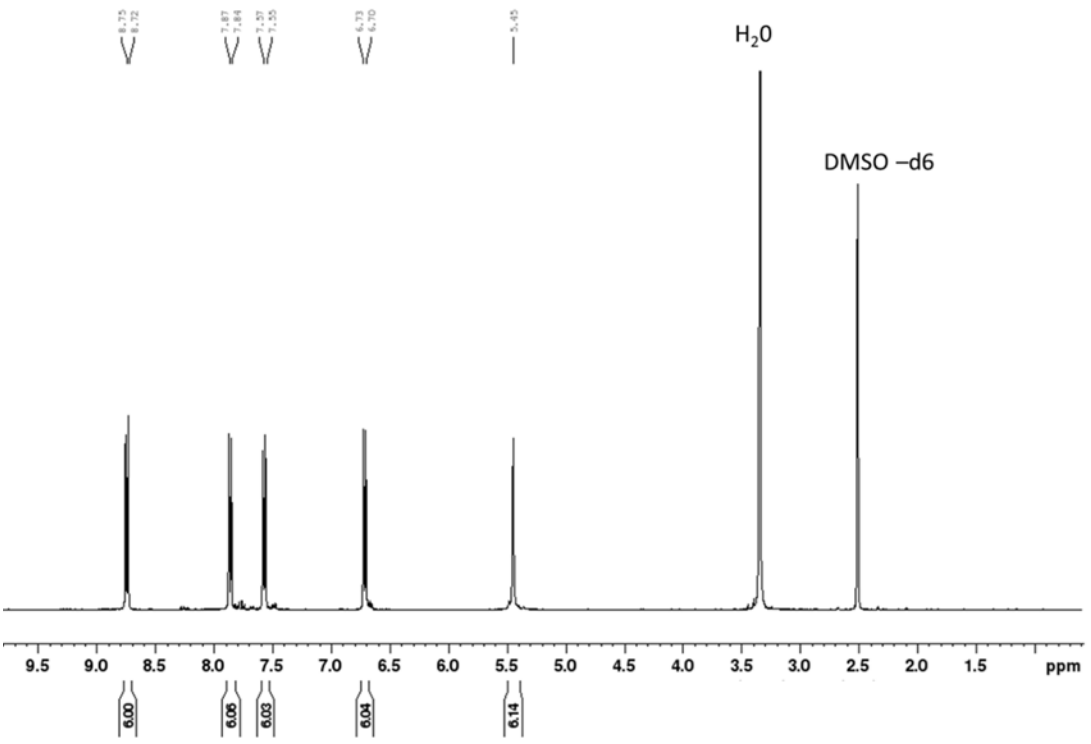

Figure 10. ^{1}H NMR (400 MHz, DMSO) of the product peaks for **TTBT.Cl**. ^{1}H NMR (400 MHz, DMSO) δ 8.73 (d, J = 8.5 Hz, 6H), 7.85 (d, J = 8.5 Hz, 6H), 7.56 (d, J = 8.5 Hz, 6H), 6.71 (d, J = 8.5 Hz, 6H), 5.46 (s, 6H).

Only one crystallisation condition investigated appeared to give single crystals of **TTBT.Cl** — this used MTF as the solvent and HCl in water as the source of HCl. The exact conditions were as follows: TTBT (5 mg) was dissolved in MTF (0.3 mL) in a vial containing a small hole, this vial was then left placed inside a larger vial containing 0.5 mL of aqueous HCl (37%) and 0.5 mL of H_2O. The outer vial was capped and left undisturbed for 16–24 h, after which small needles could be seen in the MTF solution. Attempts at isolating the single crystals from the solvent proved to be very difficult. After 16–24 h, only a small amount of **TTBT.Cl** crystals were observed, meaning that most of the **TTBT** was unreacted. Leaving the sample for longer resulted in formation of polycrystalline powders. To avoid this, the small vial containing the crystals was isolated and placed in a freezer; the crystal survived for ~3 days in the freezer, but beyond this time, the crystals re-dissolved in the solvent. Two problems were encountered in trying to collect a crystal for SCXRD; (i) the rapid evaporation of the volatile MTF solution, which resulted in fast crystallisation of the free amine, whose crystals were larger, and; (ii) the size and fragility of the **TTBT.Cl** single crystals: typically, they would shatter into smaller fragments when trying to mount the crystals. This precluded the collection of suitable SCXRD data for **TTBT.Cl** from this solvent system. SEM images of **TTBT.Cl** formed using these conditions are shown in Figure 11.

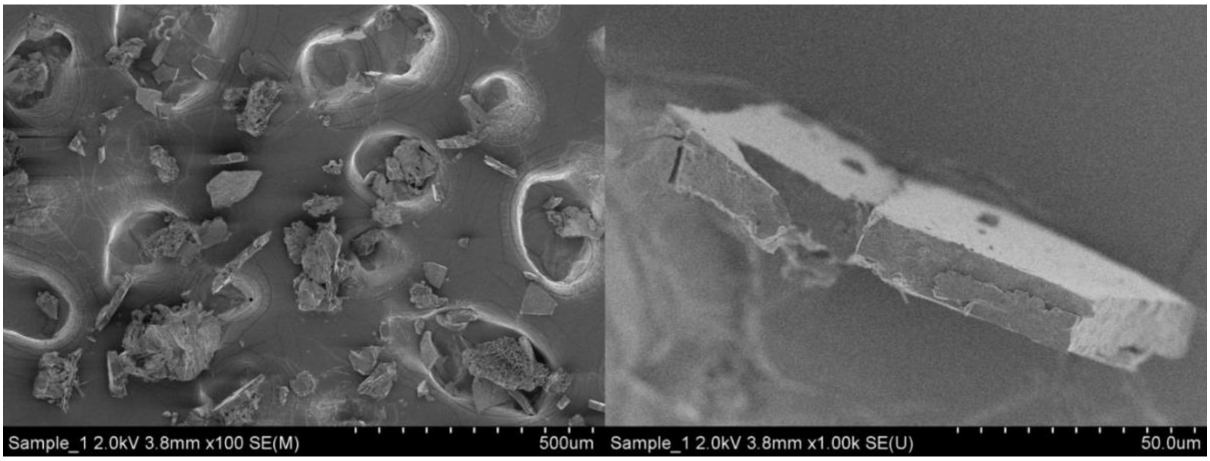

Figure 11. SEM images of **TTBT.Cl** crystals isolated from MTF and aqueous HCl; the lefthand image shows the bulk material and the righthand image shows a close-up of a single particle.

PXRD analysis for experimental and predicted TAPT.Cl, TT.Br and TTBT.Cl

Diffraction profiles were simulated for the structures most closely matching the experimental **TAPT.Cl**, **TT.Br** and **TTBT.Cl** patterns. Stack plots of the simulated and experimental patterns (Fig. 2g-i) show the similarity of the **TAPT.Cl** and **TT.Br** profiles in particular. Minor differences in the experimentally observed unit cell and predicted structure, and experimental effects such as background scattering and peak broadening tend to dominate the difference curves; hence

these are not included. To investigate the differences in the **TTBT.Cl** profiles, constrained structural refinement of **TTBT.Cl** was performed as described below.

PXRD analysis of TTBT.Cl

To reduce the possible effects of preferred orientation and improve powder averaging, PXRD data for **TTBT.Cl** were collected on a sample Debye-Scherrer geometry with a capillary spinner. The pattern has relatively low resolution with significant broadening of the diffraction peaks, probably due to a combination of the short ordered length scale, the presence of residual disordered solvent and molecular conformation. It proved challenging to identify and fit more than thirteen individual peaks in the pattern to perform autoindexing. A set of trigonal and hexagonal cells were identified by the indexing routine as candidates. These cells indicated a lattice repeat of approximately 27.4 Å within the layers and an intralayer distance of ~3.9 Å; these dimensions were consistent with the molecular structure of **TTBT.Cl**. However, the profile can be also indexed using a monoclinic unit cell, similar to lowest energy structure on the CSP landscape (Figure 2c,f in main text), and Le Bail refinement of this cell with the *C*2/*c* space group affords lattice parameters of a = 27.581(3), b = 47.314(5), c = 8.0051(9) Å, β = 97.61(1)°, V = 10355(2) Å^3. It is possible that the true symmetry of the **TTBT.Cl** structure is higher than in the predicted structure, due to the equivalence of the biphenyl substituents allowing threefold rotational molecular symmetry, noting that the CSP calculations were only performed in the 11 most common space groups.

The monoclinic unit cell and a molecular fragment consisting of the full TTBT molecule with fixed geometry and three chloride ions was used as the basis for simulated annealing structure solution. The *y*-coordinate of one Cl- ion was fixed; the fragments were otherwise allowed to translate and rotate freely within the cell. The Fourier difference maps for the initial solutions indicated significant electron density in the channels, which was inferred to be due to residual water. The calculation was repeated with the inclusion of up to six oxygen atom positions with variable occupancies. The best solution was used as a basis for structural refinement (Extended Data Fig. 3c) in which the position and orientation of the TTBT molecule were refined, in addition to the chloride and oxygen atom positions. The oxygen occupancies refined to approximately five water molecules per TTBT. Due to the low resolution of the data, individual atomic positions were not refined and the molecular conformation was fixed; hence it was not possible to explore the symmetry of the TTBT molecule further to investigate its compatibility with higher symmetry space groups. A single isotropic displacement parameter was refined for the TTBT molecule with one displacement parameter for all chloride ions. Oxygen displacement parameters were fixed at 0.05 Å^2. The refined structure confirmed that

the packing arrangement of the TTBT molecule and chloride ions are consistent with the lowest energy CSP structure.

Stability of salts to activation

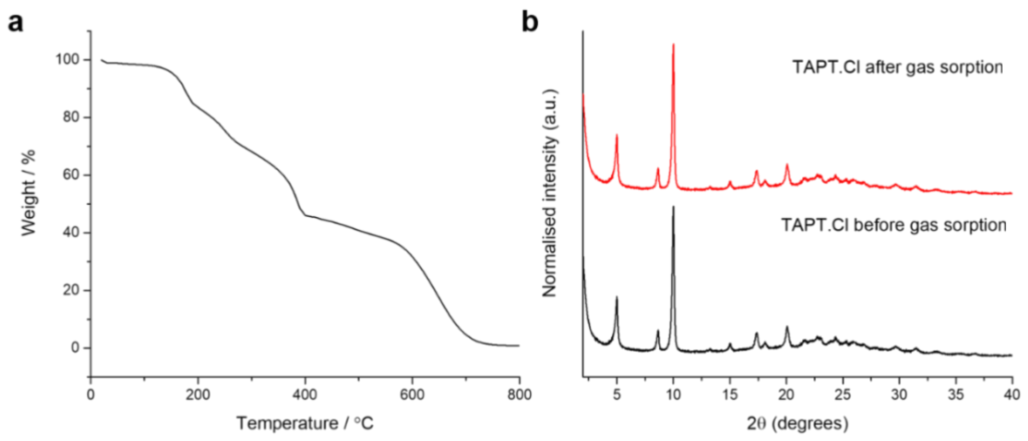

Figure 12. a. TGA trace of **TAPT.Cl** after heating under vacuum at 110 ˚C for 16 h. **b**, PXRD of the **TAPT.Cl** sample after activation and before gas sorption experiments (red) and after the gas sorption experiments (black) showing the stability of the framework to desolvation.

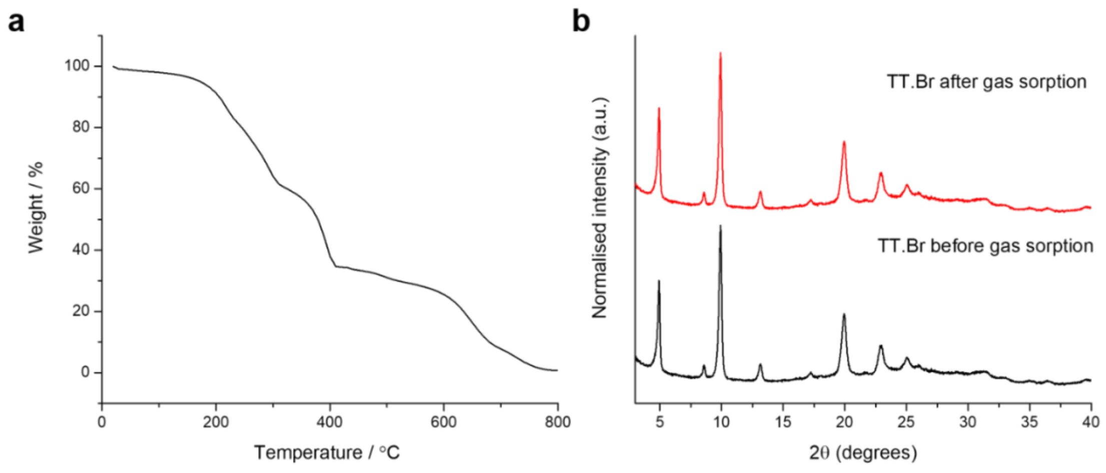

Figure 13. a. TGA trace of **TT.Br** after heating under vacuum at 110 ˚C for 16 h. **b**, PXRD of the **TT.Br** sample after activation and before gas sorption experiments (red) and after the gas sorption experiments (black) showing the stability of the framework to desolvation.

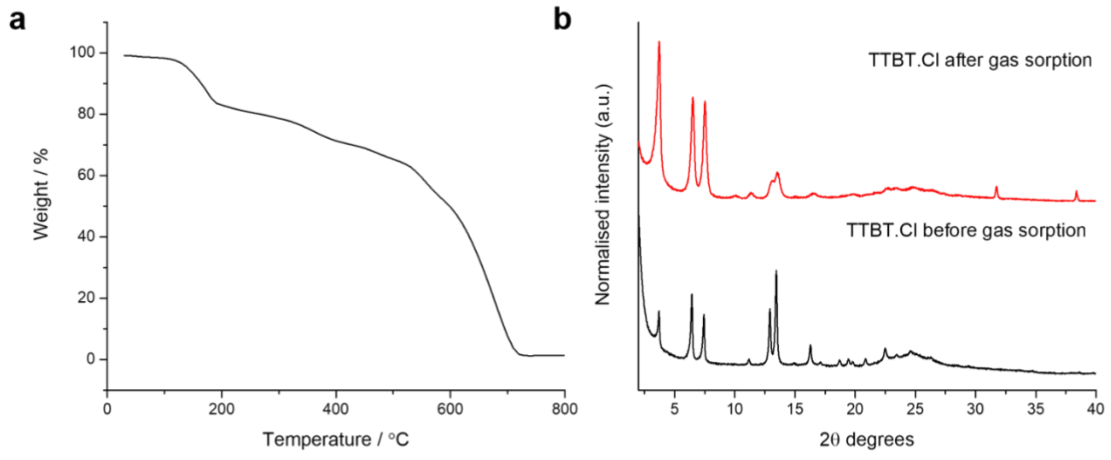

Figure 14. a. TGA trace of **TTBT.Cl** after heating under vacuum at 110 ˚C for 16 h. **b**, PXRD of the **TTBT.Cl** sample after activation and before gas sorption experiments (red) and after the gas sorption experiments (black). After the gas sorption experiments, which involve a further on-port degassing step, there is a decrease in the intensity of the Bragg peaks, suggest a loss of order under these activation / adsorption conditions that was not apparent for **TATP.Cl** (Figure 13b) or **TT.Br** (Figure 14b). The reduces the CO_2 sorption in this material (Extended Data Fig. 9). By contrast, as discussed in the main text and in Section 4 here, **TTBT.Cl** is robust over repeated iodine sorption / desorption cycles (Figure 4c) and it exhibits the highest iodine uptake of the salt frameworks studied here (248 wt. %), suggesting that the framework stability depends on the specific activation, sorption, and regeneration conditions.

Section 3. Single crystal refinement details

Table 3. Single crystal refinement data for **TAMP.Cl_P1** and **TAMP.Cl_P2**

Molecule	**TAPM.Cl_P1**	**TAPM.Cl_P2**
λ [Å]	0.71073	0.71073
Collection Temperature	100 K	100 K
Formula	$C_{25}H_{28}N_{44}$(Cl);	3.2(Cl), 0.8(C_{25} H_{28} N_4), 1.6(C H_4 O), 0.2(O):
M_r [g mol^{-1}]	64.28	529.51
Crystal Size [mm]	0.065 x 0.024 x 0.02	0.069 x 0.027 x 0.02
Crystal System	Tetragonal	Monoclinic
Space Group	*P*4$_2$/*nmc*	*C12/c1*
a [Å]	13.2945(5)	12.4607(4)
b [Å]		20.2943(7)
c [Å]	7.5715(4)	11.9466(4)
α [°]		
β [°]	90	101.801(4),
γ [°]		
V [Å^3]	1338.22(12)	2957.21(17)
Z	16	5
D_{calcd} [g cm^{-3}]	1.276	1.487
μ [mm^{-1}]	0.461	0.313
F(000)	524	524
2θ range [°]	2.166 – 30.161	1.195– 30.382
Reflections collected	5172	10396
Independent reflections, R_{int}	949, 0.0287	3589, 0.1253
Obs. Data [$I > 2\sigma(I)$]	781	781
Data /restraints / parameters	949/ 0 / 46	3589 / 2 / 241
Final R_1 values ($I > 2\sigma(I)$)	0.0357	0.0357
Final R_1 values (all data)	0.0447	0.0755
Final w$R(F_2)$ values (all data)	0.1036	0.2007
Goodness-of-fit on F^2	1.035	1.067
Largest difference peak and hole [e.A^{-3}]	0.459 / -0.223	1.032 / -0.829
CCDC	2308598	2308599

Table 4. Single crystal refinement data for **TAMP.Br_P1** and **TAPT.Cl**

Molecule	**TAPM.Br_P1**	**TAPT.Cl**
λ [Å]	0.71073	0.6889
Collection Temperature	113 K	100 K
Formula	C_{25} $H_{27.25}$ N_4, 4(Br)	3(Cl), C_{24} H_{24} N_3, 1.25[C_6H_5Cl], 1.5[H_2O];
M_r [g mol^{-1}]	238.78	628.52
Crystal Size [mm]	0.2 x 0.2 x 0.23	0.02 x 0.02 x 0.01
Crystal System	Trigonal	Trigonal
Space Group	*P1*	*P3m1*
a [Å]	11.2877(5)	35.3856(2)
b [Å]	11.7704(5)	
c [Å]	11.9638(5)	8.17500(10)
α [°]	90.265(3)	
β [°]	107.249(4)	90
γ [°]	114.896(4)	120
V [Å^3]	1361.50(11)	8864.85(15)
Z	2	12
D_{calcd} [g cm^{-3}]	1.718	1.413
μ [mm^{-1}]	4.439	0.422
F(000)	692	3930
2θ range [°]	2.106 – 30.3553	1.116– 27.339
Reflections collected	16628	144458
Independent reflections, R_{int}	6616, 0.0348	7685, 0.0797
Obs. Data [$I > 2\sigma(I)$]	5147	5263
Data /restraints / parameters	6616 / 0 / 302	7685/ 245 / 329
Final R_1 values ($I > 2\sigma(I)$)	0.0322	0.0797
Final R_1 values (all data)	0.0503	0.0962
Final w$R(F_2)$ values (all data)	0.067	0.2710
Goodness-of-fit on F^2	1.034	1.116
Largest difference peak and hole [e.A^{-3}]	0.855 / -0.854	0.706 / -0.440
CCDC	2308596	2308662

Section 4. Iodine capture experiments

These porous salt frameworks were found to absorb greater quantities of iodine vapour than most MOFs studied so far (see Refs. 9–12 in main text and in particular review article in Ref. 43). For additional reviews on iodine capture in other classes of porous solids, see Section 6 (Supplementary Refs. R1–R5).

Iodine capture / desorption in TAPM.Cl_P1

TAPM.Cl_P1 is dense and non-porous, and it absorbs almost no iodine (Figure 4a, main text); as such, the weight losses observed in Figure 15 result from thermal decomposition of the **TAPM.Cl_P1** crystals.

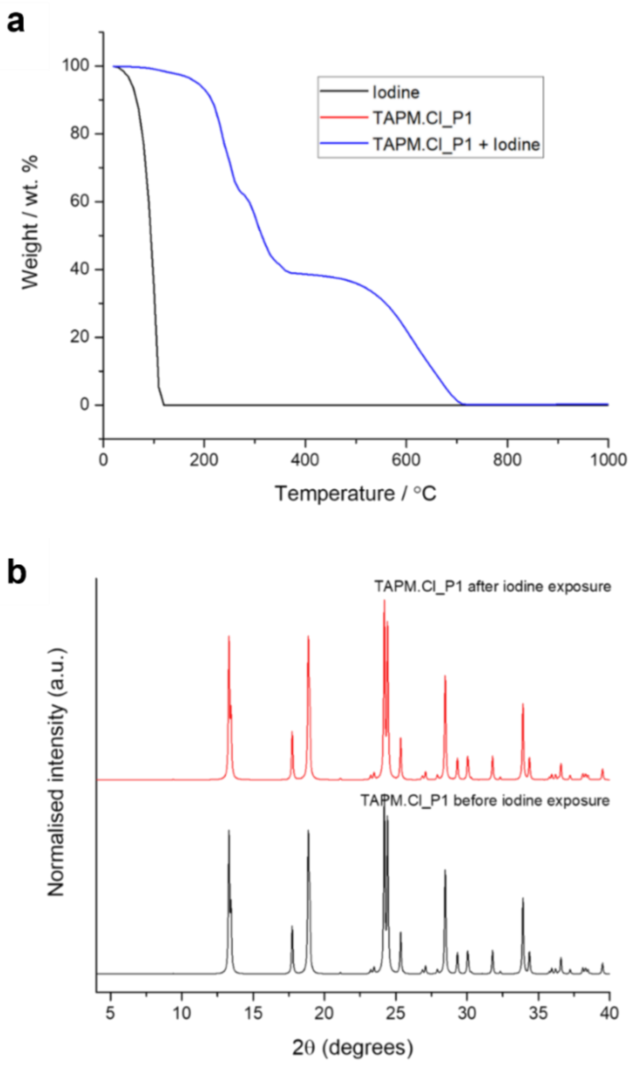

Figure 15. **a**, TGA trace of pure iodine (black) and both **TAPM.Cl_P1** before and after iodine exposure (red and blue); note that these two traces are completely superimposed. **b**, PXRD pattern for **TAPM.Cl_P1** before and after iodine exposure showing no changes.

Iodine capture / desorption in TAPT.Cl

The porous **TAPT.Cl** structure absorbs 211 wt. % iodine prior to desorption during TGA (Figure 4a, main text).

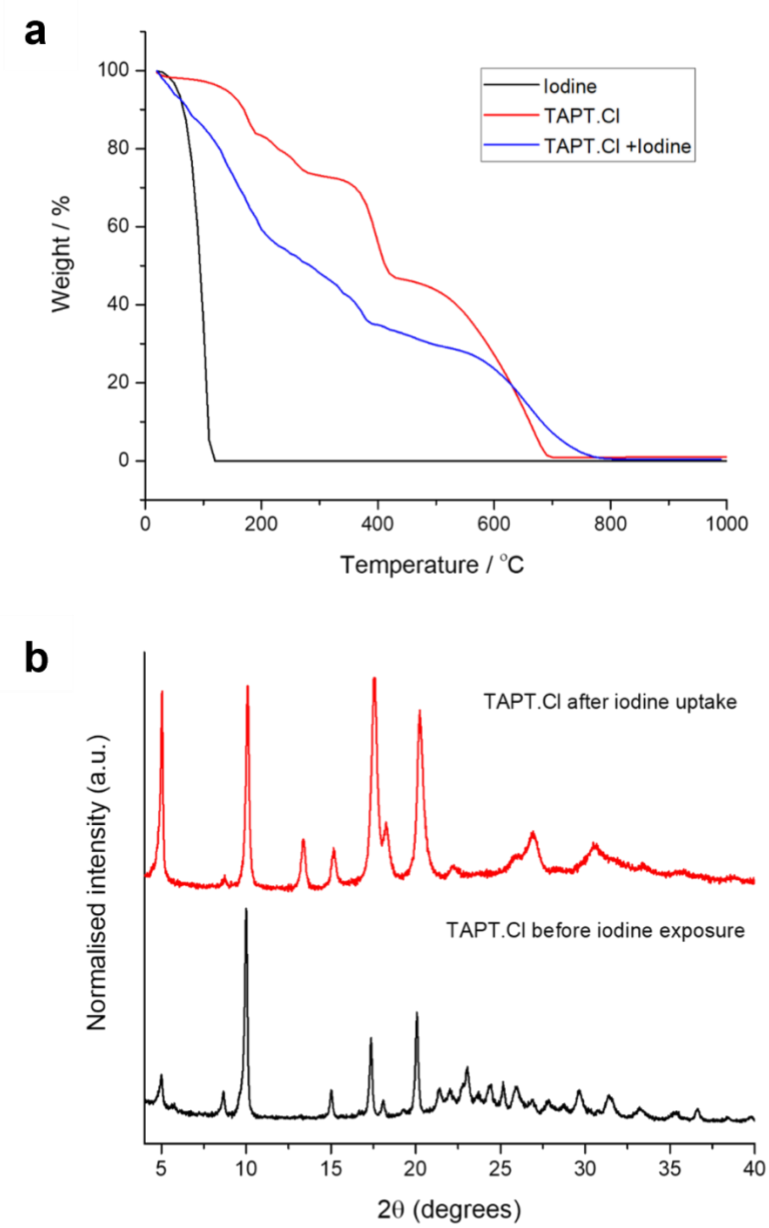

Figure 16. a, TGA traces for pure iodine (black) and **TAPT.Cl** both before (red) and after (blue) iodine exposure. **b**, PXRD patterns for **TAPT.Cl** before and after iodine exposure, suggesting changes in the structure. We would expect significant changes in the PXRD pattern, given that 211 wt. % iodine is absorbed: this highly scattering species would influence the PXRD pattern, particularly if it is ordered in the pores, even if the basic framework topology was retained.

Iodine capture / desorption in TT.Br

Like **TAPT.Cl**, the porous **TT.Br** structure absorbs significant quantities of iodine (213 wt. %) prior to desorption during TGA (Figure 4a). The time-dependent absorption profiles for these two crystalline frameworks are almost identical and reproducible over 5 separate repeats (see **Methods** in main text); that is, **TAPT.Cl** and **TT.Br** are isostructural, as predicted, and their iodine capture and release properties are therefore very similar.

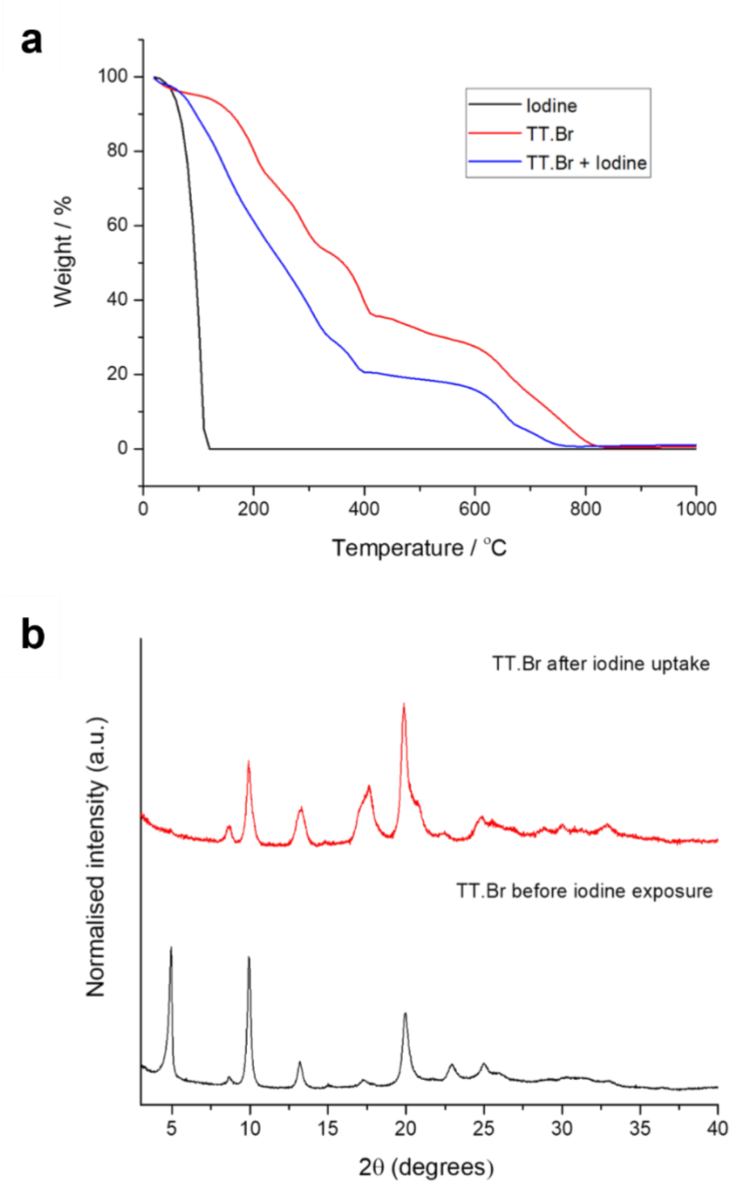

Figure 17. **a**, TGA traces for pure iodine (black) and **TT.Br** both before (red) and after (blue) iodine exposure. **b**, PXRD patterns for **TT.Br** before and after iodine exposure suggesting changes in the structure. We would expect significant changes in the PXRD pattern, given that 213 wt. % iodine is absorbed: this highly scattering species would influence the PXRD pattern, particularly if it is ordered in the pores, even if the basic framework topology was retained.

Iodine capture / loss in TTBT.Cl

The porous **TTBT.Cl** structure absorbs 248 wt. % iodine prior to desorption during TGA (Figure 4a, main text).

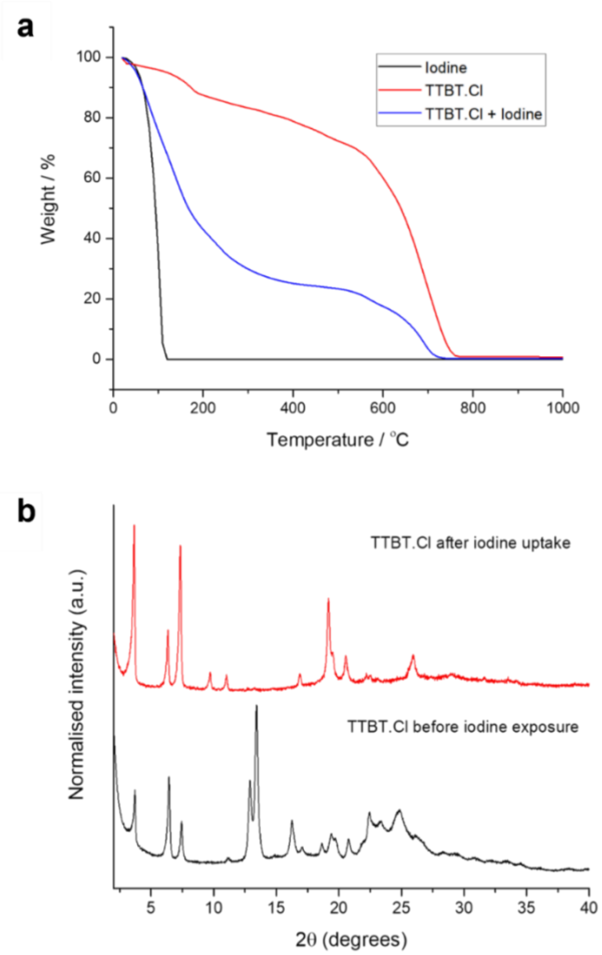

Figure 18. **a**, TGA traces for pure iodine (black) and **TTBT.Cl** both before (red) and after (blue) iodine exposure. **b**, PXRD patterns for **TTBT.Cl** before and after iodine exposure suggesting changes in the structure. We would expect that there could be significant structural changes to the **TTBT.Cl** framework and associated changes in the PXRD pattern, given that 248 wt. % iodine is absorbed. Moreover, even if the framework topology were unchanged, iodine is a good x-ray scatterer, and any ordered iodine in the framework pores might be expected to affect the PXRD pattern. Significant changes to the PXRD pattern are indeed observed after iodine sorption, but we note that the original PXRD pattern is completely regenerated upon iodine removal (Figure 4d, main text) and that this can be done at least five times without any loss in iodine adsorption capacity (Figure 4c, main text), demonstrating that any structural changes in the framework are fully reversible.

Recyclability experiments for iodine capture

To test the recyclability of the salts for iodine capture, the iodine was removed from each sample and the iodine capture and release performance was measured over five cycles (*e.g.*, Figure 4c, main text). Due to the high mass of iodine captured in each system and the slow release using vacuum alone, chloroform ($CHCl_3$) was first used to remove the bulk of the iodine (Figure 19) before the final traces of iodine were removed at 70 °C under vacuum for 16 h.

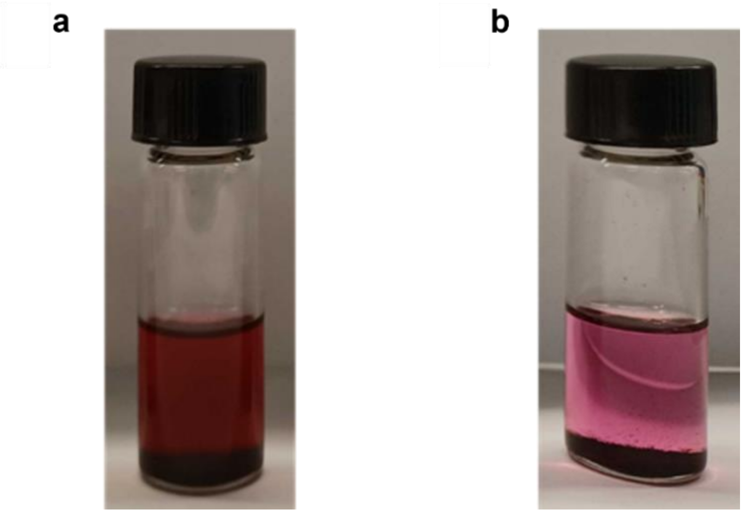

Figure 19. **a**, **TAPT.Cl** sample containing iodine; image recorded 2 min after the first addition of $CHCl_3$, showing almost instant iodine release. **b**, The same sample after 2 days of $CHCl_3$ solvent swaps, at which point the colour in the solvent is much paler. The iodine is rather difficult to extract / release from these salts, but this should be advantageous for radioiodine capture applications.

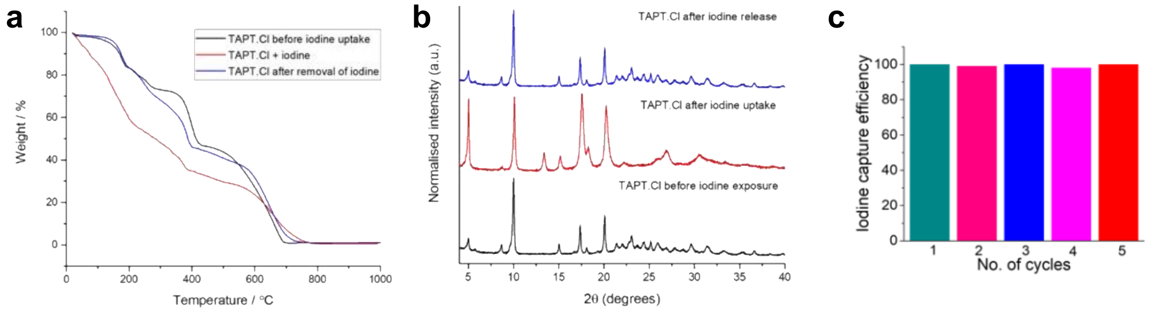

Figure 20. a, TGA trace of **TAPT.Cl** with iodine (red), and before (black) and after (blue) iodine removal. **b**, PXRD pattern for **TAPT.Cl** before iodine exposure, after iodine absorption, and after subsequent iodine removal that the original PXRD pattern for the salt is regenerated and the absorption process is reversible. **c**, Recyclability tests over 5 cycles for **TAPT.Cl**, where 100% efficiency is defined as the initial iodide uptake (cycle 1). Figure 4, main text, shows comparable data for **TTBT.Cl**.

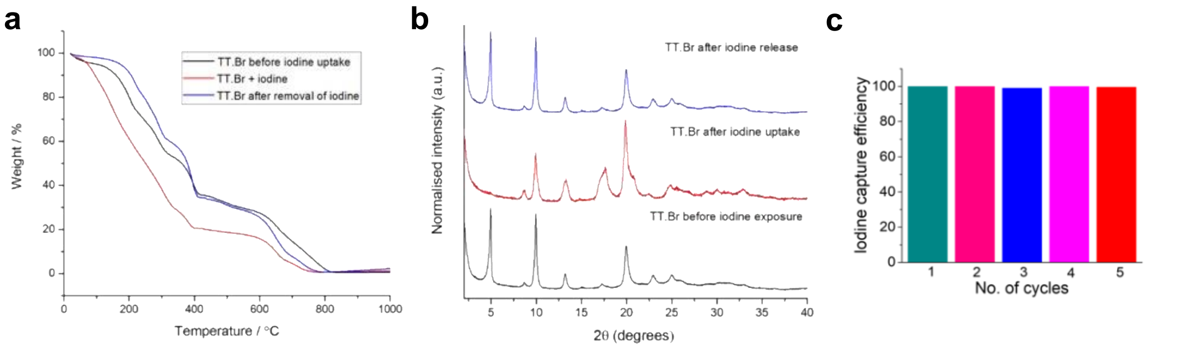

Figure 21. a, TGA trace of **TT.Br** with iodine (red), and before (black) and after (blue) iodine removal. **b**, PXRD pattern for **TT.Br** before iodine exposure, after iodine absorption, and after subsequent iodine removal that the original PXRD pattern for the salt is regenerated and the absorption process is reversible. **c**, Recyclability tests over 5 cycles for **TT.Br**, where 100% efficiency is defined as the initial iodide uptake (cycle 1). Figure 4, main text, shows comparable data for **TTBT.Cl**.

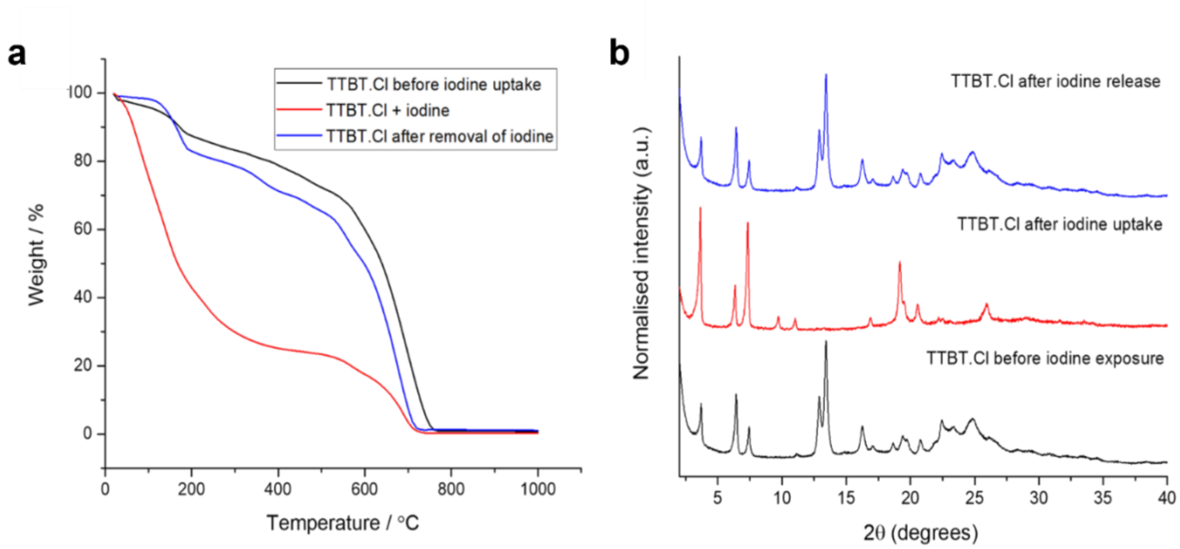

Figure 22. a, TGA trace of **TTBT.Cl** with iodine (red), and before (black) and after (blue) iodine removal. **b**, PXRD pattern for **TTBT.Cl** before iodine exposure, after iodine absorption, and after subsequent iodine removal that the original PXRD pattern for the salt is regenerated and the absorption process is reversible (see also Figure 4, main text).

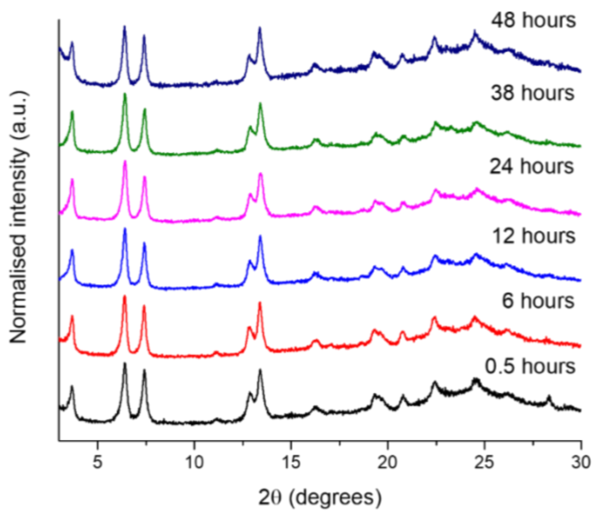

Figure S23. PXRD patterns for a porous **TTBT.Cl** sample that was submerged in water at room temperature, as sampled at different time intervals. By contrast, the porous **TAPT.Cl** framework is soluble in water, and **TT.Br** becomes amorphous upon immersion in water.

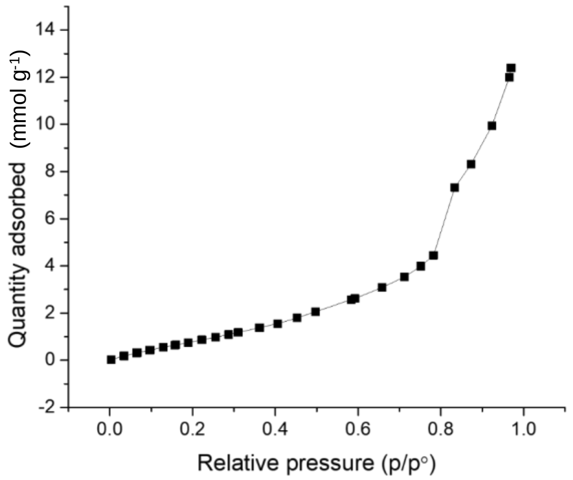

Figure S24. Water adsorption isotherm for the porous **TTBT.Cl** framework. The maximum water uptake is 12.4 mmol g^{-1}.

Section 5. Crystal structure prediction

The main details for the CSP methodology are given in the **Methods** section of the main text. Additional details are given here regarding (i) amine linker conformers, (ii) space groups, (iii) bromide potential parameters, (iv) tight-binding DFT corrections and (v) the influence of distance thresholds on energy–structure maps for ammonium halide charge adjacency.

Conformers of the amine linkers

It should be noted that the ammonium group at the end of each linker arms also has rotational flexibility, but this was fixed rigid because the energy barrier between various rotations of this group is generally < 0.3 kJ mol^{-1} in PBE0/6-311G calculations. Nevertheless, to ensure that the rotation of the amine group would not alter the CSP outcome, we performed CSPs with different rotations of the amine group at the end of the leg and found that this reproduced the same energy landscapes.

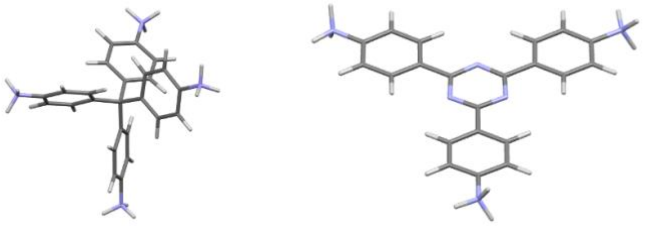

Figure 25. Conformers of **TAPM** (left) and **TT** (right) used in the CSP searches.

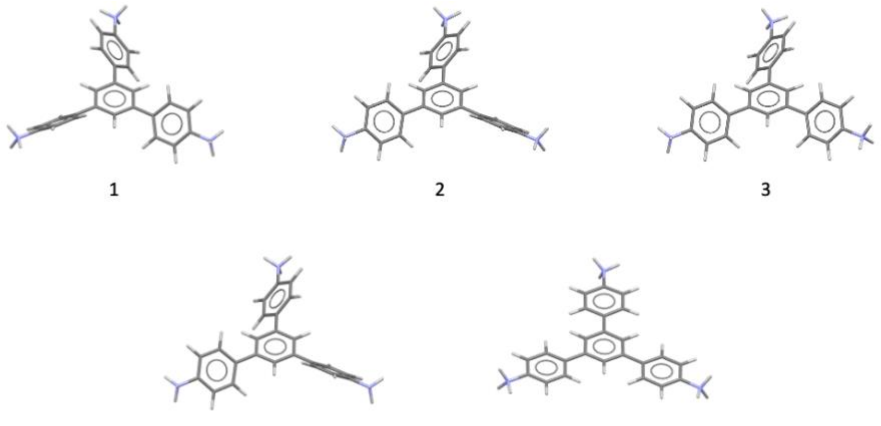

Figure 26. The four **TAPT** conformers used in the CSP searches. The hypothetical planar **TAPT** structure is also shown, but because of its dynamic instability, it was not used for crystal structure prediction.

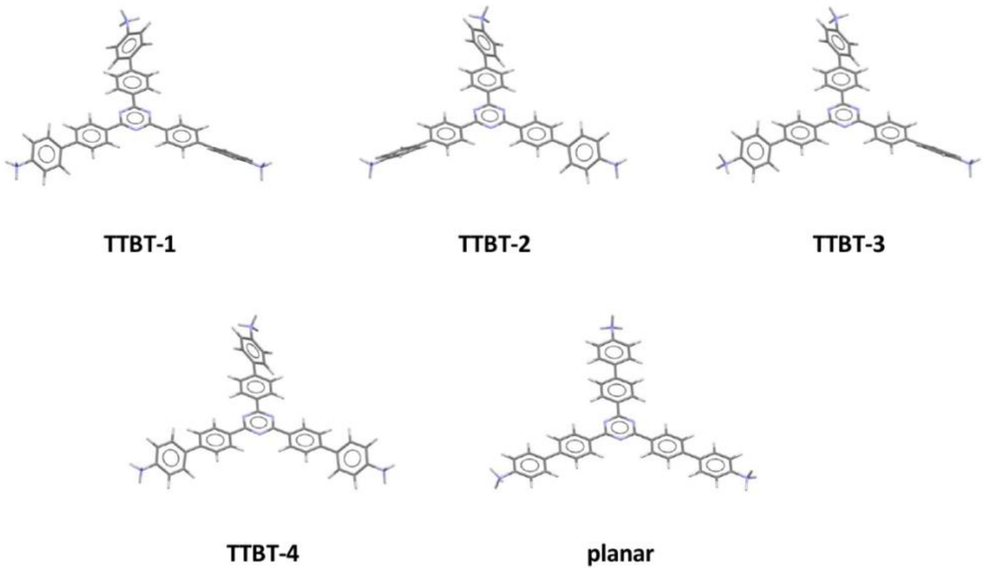

Figure 27. The four **TTBT** conformers used in the CSP searches. The hypothetical planar **TTBT** structure is also shown, but because of its dynamic instability, it was not used for crystal structure prediction.

Table 5. Relative stabilities of the four **TAPT** conformers

Conformer	Relative energy (kJ mol^{-1})
TAPT-1	0.00
TAPT-2	0.05
TAPT-3	0.15
TAPT-4	1.32

Table 6. Relative stabilities of the four **TTBT** conformers

Conformer	Relative energy (kJ mol^{-1})
TTBT-1	0.00
TTBT-2	0.28
TTBT-3	0.11
TTBT-4	0.36

Bromide potential parameters

The bromide parameters used in the work were taken from a molecular dynamics study of the thermodynamic properties of calcium apatites[R6]. To complete the set of force-field parameters, interactions between bromide ions and other atoms were determined using a series of mixing rules that were introduced with the revised version of the Williams 99 force-field[R7]. To determine the suitability of these parameters, we performed CSP on a small series of bromide salts from the Cambridge Structural Database (Figure 28). This allowed us to determine whether we could predict the known polymorphs of these salts and whether the force-field ranked them low in energy on the CSP landscape. CSP was performed using the methodology outlined in the main text, with the exception that we only sampled the space group in which the experimental polymorph was observed.

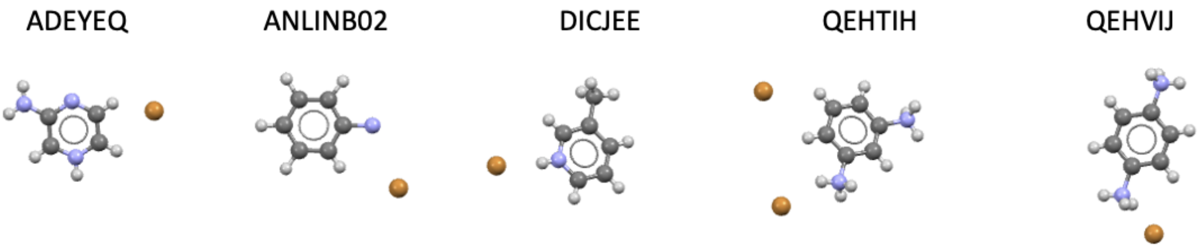

Figure 28. CCDC REFCODEs and images of the benchmark salt structures used in the bromide potential parameter tests.

In general, the force-field was able to generate the experimentally observed polymorph structures with good accuracy and rank them low in energy on the CSP landscape (Table 7). The overlay RMSDs range between 0.141 and 0.409 Å, which indicates good agreement between the CSP match and experimental polymorphs. In the case of **DICJEE,** the energy ranking of the observed polymorph was poorer than what we observed for the other salts. Its CSP match was found 10.4 kJ mol^{-1} above the global minimum structure and ranked 63rd overall. However, by expanding the CSP search into other sub-groups such as $P2_1/c$, $P\bar{1}$ and $Pna2_1$, a lower energy match with an improved overlay RMSD was found. A visual aid for these RMSD values is given in Figure 29.

Table 8. Results of the energy ranking and overlay accuracy for the CSP structures that matched the observed polymorphs of five bromide salts.

REFCODE	Space group	Number of sampled structures	Experimental match RMSD (Å)	ΔE from global minimum, (kJ mol^{-1})	Energy ranking on CSP landscape
ADEYEQ	$P\bar{1}$	10,000	0.409	+0.187	3rd
ANLINB02	$P2_1/c$	20,000	0.197	+2.67	6th
DICJEE	*Pnma*	10,000	0.254	+10.8	63rd
	$Pna2_1$	20,000	0.141	+3.48	3rd
QEHTIH	$P2_1/c$	20,000	0.357	+0.240	2nd
QEHVIJ	*P1*	10,000	0.225	+1.34	3rd

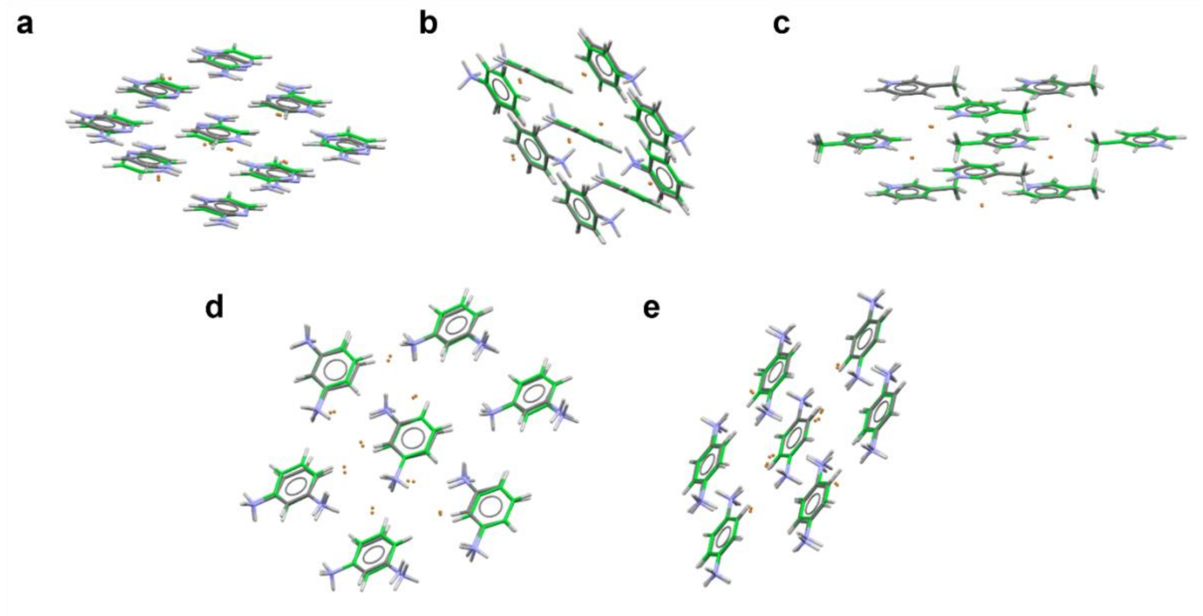

Figure 29. Crystal structure overlays of ADEYEQ, ANLINB02, DICJEE, QEHTIH, and QEHVIJ with their matching CSP structures shown in green.

Space groups sampled in CSP calculations

Trial crystal structures were generated across eleven space groups and their lattice energies were minimized until a target number of valid crystal structures was met (Table 8).

Table 8. List of space groups that were sampled in this study.

Space group name	Space group number	Number of valid structures to generate
$P1$	1	10,000
$P\bar{1}$	2	10,000
$P2_12_12_1$	19	10,000
$Pna2_1$	33	20,000
$P2_1$	4	20,000
$C2$	5	20,000
Cc	9	20,000
$Pbca$	61	20,000
$P2_1/c$	14	50,000
$C2/c$	15	50,000
$R\bar{3}$	148	50,000

Tight-binding DFT corrections

For CSPs involving the **TAPM** cation, a model of **TAPM** was constructed and optimised to the nearest local gas-phase minimum. Unlike **TAPT**, which has multiple gas-phase conformers, we found that **TAPM** only adopts one (Figure 26) due to the steric demand of the four anilinium groups around the central sp^3 carbon. After performing CSP on **TAPM.Cl**, we were able to locate two matches to the experimental polymorphs on the CSP landscape (main text, Figure 2a). However, for the predicted structure of **TAPM.Cl/P1**, there was a clear mismatch between the conformation adopted by the cation in the gas phase and its conformation in the **TAPM.Cl** crystal. To investigate this further, all structures up to a relative total energy of 100 kJ mol^{-1} were re-optimised via tight-binding DFT (see **Methods**, main text) resulting in a new CSP landscape (Figure 30). After allowing the cations intramolecular degrees of freedom to relax within the crystal environment, we were able to improve the matches to both experimental polymorphs (Figure 31).

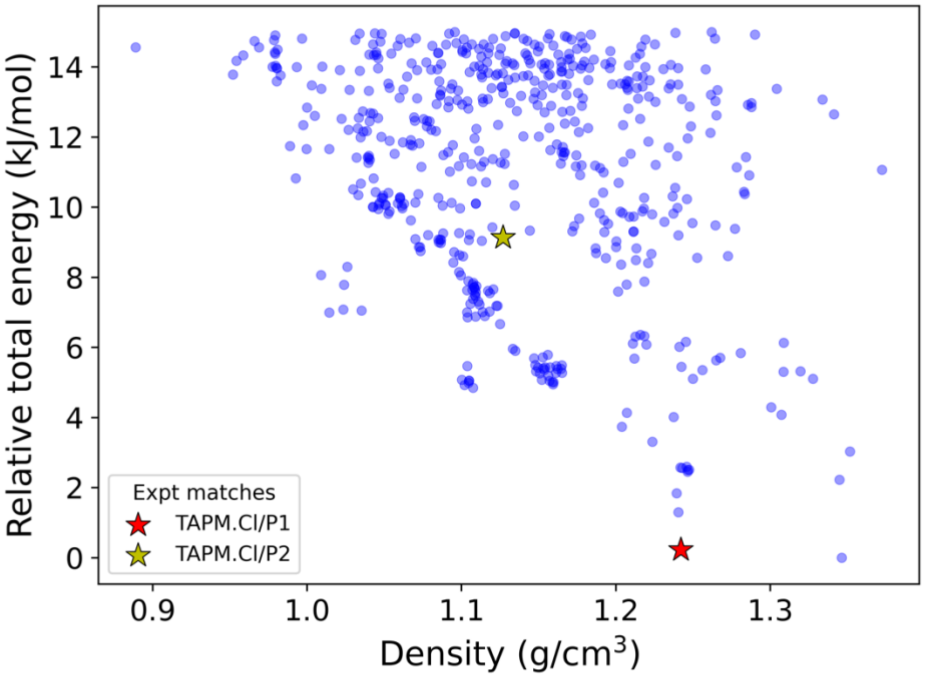

Figure 30. CSP landscape for **TAPM.Cl** after tight binding DFT-based optimisations of all crystal structures within 100 kJ mol^{-1} of the global energy minimum.

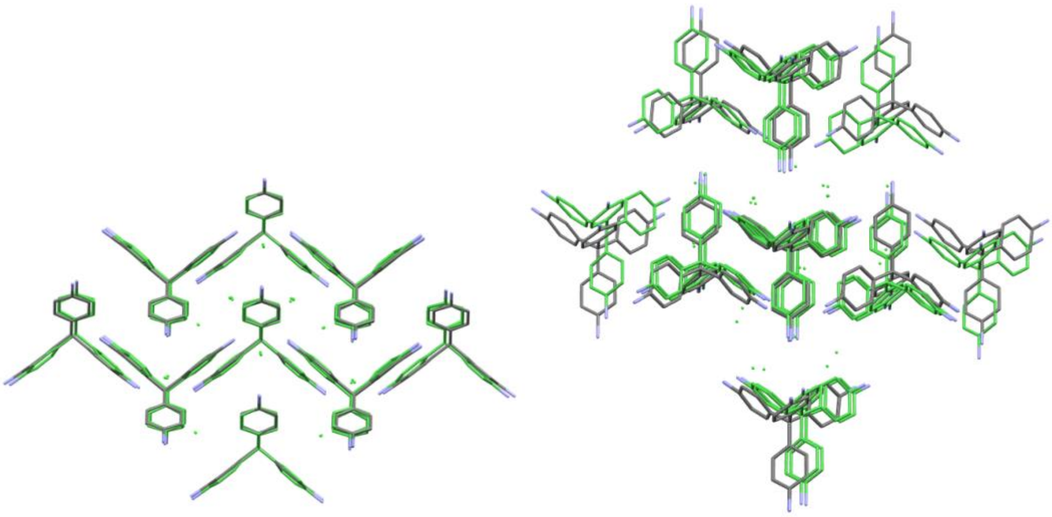

Figure 31. Overlay images for experimental structures (coloured by element) and DFTB re-optimised structures (green) for the two experimentally accessible polymorphs: **TAPM.Cl/P1** (left) and **TAPM.Cl/P2** (right).

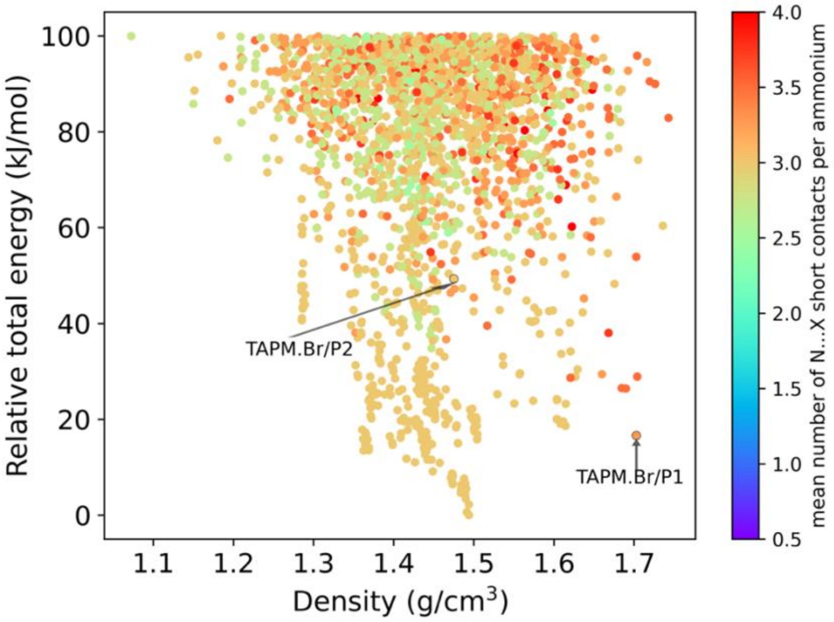

Figure 32. CSP landscape of **TAPM.Br** where each structure is coloured by the mean number of N-Br short contacts per ammonium. N-Br contacts were evaluated at a distance of 4.25 Å.

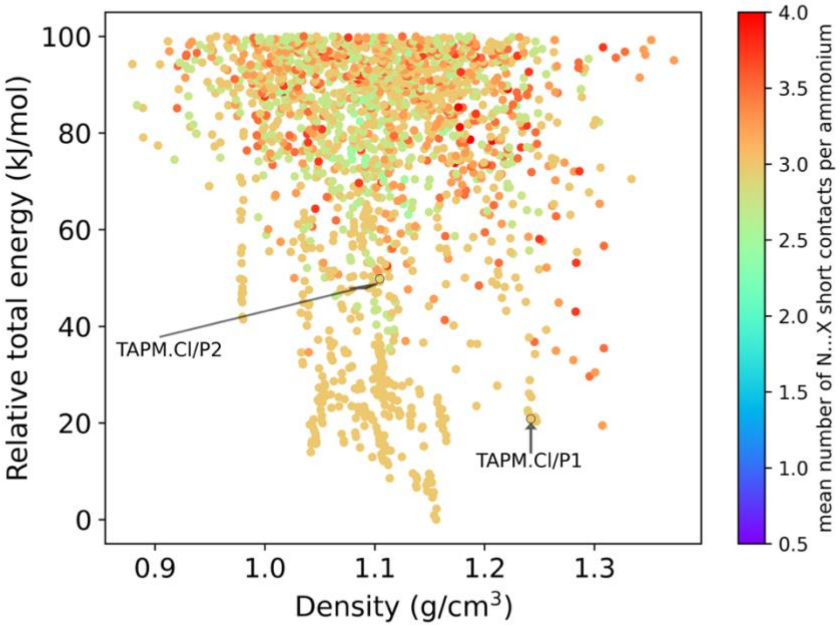

Figure 33. CSP landscape of **TAPM.Cl** where each structure is coloured by the mean number of N-Cl short contacts per ammonium. N-Cl contacts were evaluated at a distance of 4.125 Å.

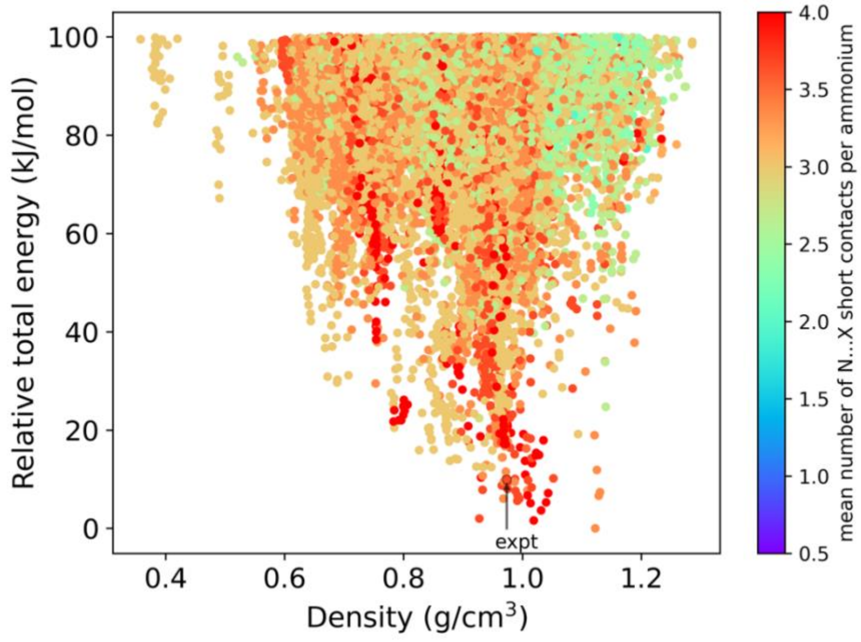

Figure 34. CSP landscape of **TAPT.Cl** where each structure is coloured by the mean number of N-Cl short contacts per ammonium. N-Cl contacts were evaluated at a distance of 4.125 Å.

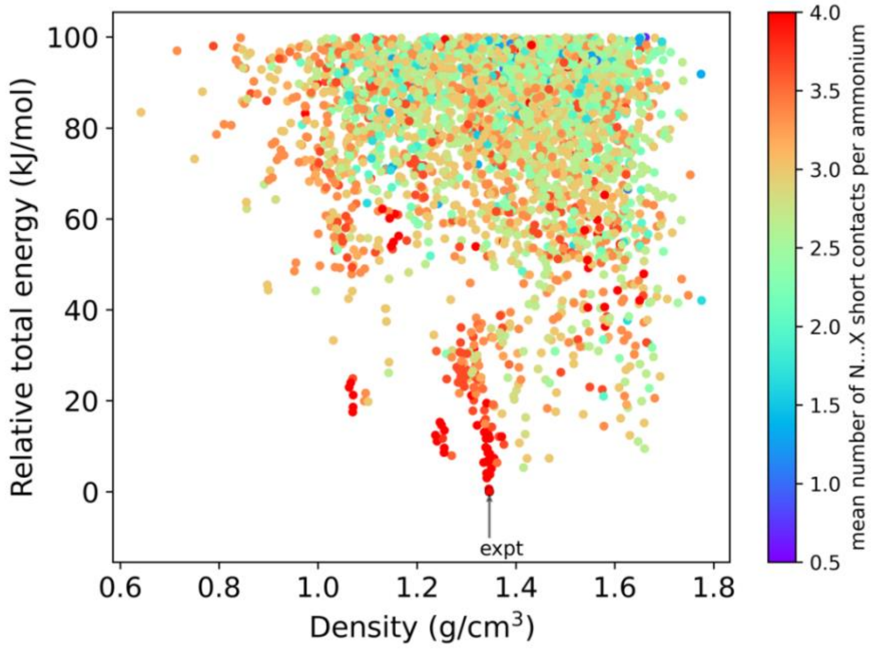

Figure 35. CSP landscape of **TT.Br** where each structure is coloured by the mean number of N-Br short contacts per ammonium. N-Br contacts were evaluated at a distance of 4.25 Å.

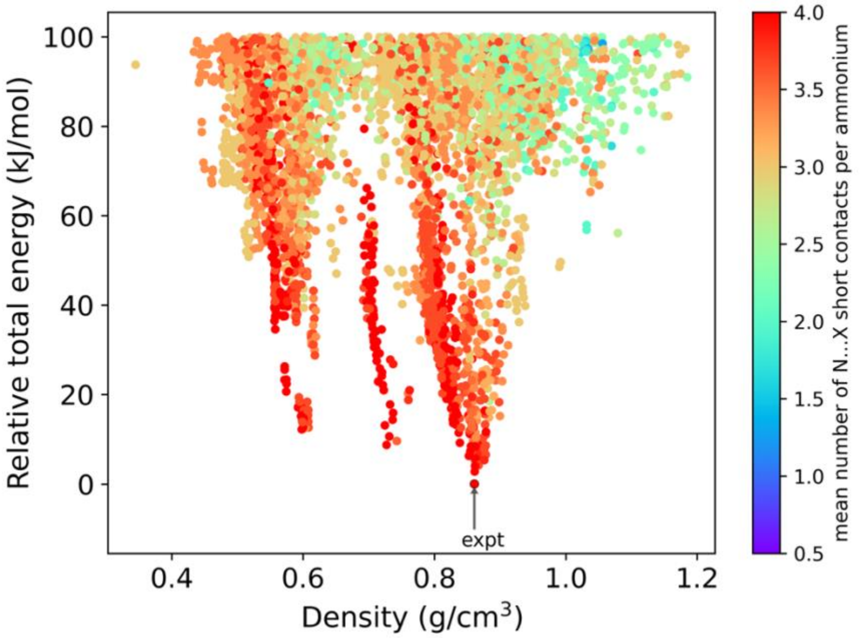

Figure 36. CSP landscape of **TTBT.Cl** where each structure is coloured by the mean number of N-Cl short contacts per ammonium. N-Cl contacts were evaluated at a distance of 4.125 Å.

Section 6. Supplementary references

R1. Yang, Y. T., Tu, C. Z., Yin, H. J., Liu, J. J., Cheng, F. X. & Luo, F., Molecular iodine capture by covalent organic frameworks, *Molecules*, **27**, 9045 (2022).

R2. Yu, Y.-N., Yin, Z., Cao, L.-H. & Ma, Y.-M., Organic porous solid as promising iodine capture materials, *J. Incl. Phenom. Macrocycl. Chem.*, **102**, 395–427 (2022).

R3. Kurisingal, J. F., Yun, H. & Hong, C. S., Porous organic materials for iodine adsorption, *J. Hazard. Mater.*, **458**, 10.1016/j.jhazmat.2023.131835 (2023).

R4. Huve, J., Ryzhikov, A., Nuoali, H., Lalia, V., Augé, G. & Daou, T. J., Porous sorbents for the capture of radioactive iodine compounds: a review, *RSC Adv.*, **8**, 29248–29273 (2018).

R5. Xie, W., Cui, D., Zhang, S. R., Xu, Y. H. & Jiang, D. L., Iodine capture in porous organic polymers and metal-organic frameworks materials, Mater. Horiz., 6, 1571–1595 (2019).

R6. Cruz, F. J. A. L. , Lopes, J. N. C. , Calado, J. C. G. & Minus da Piedade, M. E., A molecular dynamics study of the thermodynamic properties of calcium apatites. 1. hexagonal phases, *J. Phys. Chem. B*, **109**, 51, 24473–24479 (2005).

R7. Pyzer-Knapp, E. O., Thompson, H. P. G. & Day, G. M., An optimized intermolecular force field for hydrogen-bonded organic molecular crystals using atomic multipole electrostatics, *Acta Cryst*., **B72**, 477–487 (2016).
